# Biocompatible DNA/5-Fluorouracil-Gemini Surfactant-Functionalized Gold Nanoparticles as Promising Vectors in Lung Cancer Therapy

**DOI:** 10.3390/pharmaceutics13030423

**Published:** 2021-03-21

**Authors:** Rosa M. Giráldez-Pérez, Elia Grueso, Inmaculada Domínguez, Nuria Pastor, Edyta Kuliszewska, Rafael Prado-Gotor, Francisco Requena-Domenech

**Affiliations:** 1 Departments of Cellular Biology, Physiology and Immunology, University of Córdoba, 14014 Córdoba, Spain; v02redof@uco.es; 2 Department of Physical Chemistry, University of Seville, 41012 Seville, Spain; pradogotor@us.es; 3 Department of Biology and Cellular Biology, University of Seville, 41012 Seville, Spain; idomin@us.es (I.D.); npastor@us.es (N.P.); 4 Chemtra Company, 47-300 Krapkowize, Opolskie, Poland; edyta.kuliszewska@interia.pl

**Keywords:** chemotherapy, nanomedicine, gold nanoparticles, DNA, gemini surfactants, 5-fluorouracil, drug carriers

## Abstract

The design and preparation of novel nanocarriers to transport cancer drugs for chemotherapy purposes is an important line of research in the medical field. A new 5-fluorouracil (5-Fu) transporter was designed based on the use of two new biocompatible gold nanosystems: (i) a gold nanoparticle precursor, Au@16-Ph-16, stabilized with the positively charged gemini surfactant 16-Ph-16, and (ii) the compacted nanocomplexes formed by the precursor and DNA/5-Fu complexes, Au@16-Ph-16/DNA–5-Fu. The physicochemical properties of the obtained nanosystems were studied by using UV–visible spectroscopy, TEM, dynamic light scattering, and zeta potential techniques. Method tuning also requires the use of circular dichroism, atomic force microscopy, and fluorescence spectroscopy techniques for the prior selection of the optimal relative Au@16-Ph-16 and DNA concentrations (R = C_Au@16-Ph-16_/C_DNA_), biopolymer compaction/decompaction, and 5-Fu release from the DNA/5-Fu complex. TEM experiments revealed the effective internalization of the both precursor and Au@16-Ph-16/DNA–5-Fu-compacted nanosystems into the cells. Moreover, cytotoxicity assays and internalization experiments using TEM and confocal microscopy showed that the new strategy for 5-Fu administration enhanced efficacy, biocompatibility and selectivity against lung cancer cells. The differential uptake among different formulations is discussed in terms of the physicochemical properties of the nanosystems.

## 1. Introduction

Cancer is a leading cause of death worldwide, exerting tremendous physical, emotional, and financial strain on individuals, families, communities, and health systems [1,2,3]. Chemotherapy is used as a treatment against malignant neoplasia’s principle multiple side effects and derived sequelae. In this sense, one of the most widely used strategies to prevent the progression of cancers is the inactivation of cell death pathways and the elimination of damaged cells [4].

However, advances in new therapies are essential to find ways to selectively act on fast-growing malignant cells that do not affect healthy cells [5]. Significant progress has been made in nanomedicine to facilitate drug delivery and improve its adsorption, directly acting on the site of damage and reducing multiple side effects in patients [6,7]. Furthermore, the possibility of the controlled manipulation of material at the nanometer-length scale, facile functionalization with different drugs, improved solubility, permeability, and targetability increase the impact of nanotechnology on cancer research [8]. In fact, nanoparticles have been shown to be useful as carriers to treat a variety of cancers in chemotherapy, such as pancreatic cancer and breast cancer [9,10,11], non-small cell lung cancer (NSCLC) [12,13], gastrointestinal cancer [14], hepatic cancer [15], colorectal cancer [16,17], ovarian carcinoma [10,18,19], and glioblastoma multiforme [20]. 

More specifically, noble metal nanoparticles have demonstrated potential biomedical applications due to their remarkable role in the detection and treatment of severe diseases like cancer [21]. Due to their small size, nanoparticles can easily interact with biomolecules both on the surface and inside cells, and they can provide protection to biomolecules like DNA/RNA against enzymatic degradation [22]. For instance, silver nanoparticles are some of the most investigated metallic nanoparticles for delivery purposes due to their recognized antimicrobial properties, antiviral potential, good penetration into malignant cells, and effective anticancer activity [23]. However, among noble metal nanoparticles, gold nanoparticles (AuNPs) stand out due to their unique optical properties, excellent biocompatibility, and low toxicity [24]. Furthermore, AuNPs are smaller, with an average diameter of 50 nm [25], which is quite consistent for entry into any type of cancer cell. AuNPs synthesized by the bottom-up approach are considered to be more suitable for biomedical applications due to their relatively uniform shapes and sizes [26]. In fact, in these strategies, the reduction of Au^+3^ salts in the presence of various reducing and stabilizing agents passivates the nanoparticle’s surface, thereby preventing further aggregation [27]. In this sense, using cationic gemini surfactants as coating agents improves AuNPs’ performance in showing good antimicrobial activity, low cytotoxicity, and good biodegradability [28,29]. These types of surfactant, composed of two hydrophobic chains and two hydrophilic head groups covalently attached by a spacer, have been demonstrated to be efficient complexing and compacting agents for biopolymers for improving gene transport to the cell [30,31,32,33,34]. Specifically, 16-Ph-16 stands out due to its capacity to induce reversible DNA compaction [33]. Moreover, a biopolymer coating like DNA not only improves the biocompatibility of the nanosystem but also the colloidal stability and systemic circulation time [34,35].

One of the most common drugs used for chemotherapy is 5-fluorouracil (5-Fu), which is noteworthy given its great versatility in the treatment of several solid tumors, including lung cancers [36]. 5-Fu is an analog of uracil with a fluorine atom. Its primary mechanism of action consists of mediating the inhibition of the nucleotide synthetic enzyme thymidylate synthase (TS) [37]. However, toxic effects are caused by the misincorporation of fluoronucleotides into RNA and DNA [37]. Its serious side effects, including severe gastrointestinal toxicity, hematologic disturbance, and heart toxicosis, limit its clinical benefits [38]. In this sense, a new strategy for improving 5-Fu efficiency was developed by Liszbinski and coworkers based on 5-Fu loading on gold nanoparticles coated with epidermal growth factor receptors antibodies (anti-EGFR) for colorectal cancer treatment [39]. Other recent approaches for improving the colon delivery of 5-fluoracyl include the preparation and optimization of 5-Fu-loaded biogenic gold nanoparticles with pluronic-based coatings [40]. However, some problems related to 5-Fu drug resistance compromise its effectiveness in lung cancer therapy [41]. Therefore, it is necessary to develop new vectors based on nanoparticles to overcome this issue.

In a previous work, Au@16-Ph-16 nanoparticles were effectively covered with new Micro-RNA (miR)-generating nanomaterials for possible therapeutic applications related to obesity and insulin resistance treatments [34]. In fact, a reduced level of miR-21 might be associated with obesity and its related metabolic traits such as hyperinsulinemia [42]. In the present work, the Au@16-Ph-16 precursor was covered with DNA–5-Fu complexes to form new nanomaterials for possible application in lung cancer treatment. The combined use of 16-Ph-16 cationic surfactant as a DNA-compacting and -complexing ligand together with a DNA biopolymer to ensure biocompatibility and effective drug complexation contributed to improving the therapeutic efficacy of the 5-Fu. Moreover, with respect to the previous Au@16-Ph-16/miR-21 nanosystem, the possibility of inducing a reversible DNA conformational change in the new nanocomplexes was remarkable; that is, the Au@16-Ph-16 precursor specifically induced the reversible DNA–5-Fu compaction at the appropriate Au@16-Ph-16/DNA molar ratio. Therefore, Au@16-Ph-16/DNA–5-Fu nanosystems delineate the new avenues for a fresh strategy to induce 5-Fu drug release from compacted nanosystems in the target site upon precursor addition. Importantly, the obtained results show the effective internalization and moderate selectivity of the nanosystems against cancer cells. All these findings constitute a new goal in the development of more efficient nanosystems for cancer drug transfection into cells.

## 2. Materials and Methods

### 2.1. Materials

All chemicals were analytic-grade reagents and were used without further purification. Sodium tetrahydridoborate (NaBH4) was purchased from Panreac Química S.L.U. (Barcelona, Spain); DNA, 5-fluorouracil (5-Fu), hydrogen tetrachloroaurate (III) trihydrate, sodium cacodylate, and 3-aminopropyltriethoxilane (APTES) were purchased from Sigma-Aldrich-Merck KGaA (Darmstadt, Germany); DNA was used without further purification since preliminary experiments showed that purification did not produce changes in experimental results. The absorbance ratio of the DNA stock solutions at 260 and 280 nm was monitored and found to be between 1.8 and 1.9 (A_260_/A_280_ = 1.87), indicating no protein contamination [43]. An agarose gel electrophoresis test using ethidium bromide indicated that the average number of base pairs per DNA molecule was above 10,000 bp. To obtain the DNA concentrations in base pairs (C_DNA_), double stranded DNA concentrations (ds-DNA) were spectrophotometrically determined at 260 nm from 13,200 M^−1^·cm^−1^ DNA molar absorptivity [44]. All the solutions were prepared with de-ionized and autoclaved water (conductivity being less than 10^−6^ S·m^−1^) at a fixed ionic strength (I) of 1.63 mM. The total concentrations of the DNA polynucleotide, 5-Fu drug, 16-Ph-16 gemini surfactant, gold nanoparticles functionalized with 16-Ph-16 (Au@16-Ph-16), and the compacted nanosystem (Au@16-Ph-16/DNA–5-Fu) in a working solution are now referred to as C_DNA_, C_5-Fu_, C_16-Ph-16_, C_Au@16-Ph-16_, and C_Au@16-Ph-16/DNA–5-Fu_, respectively.

Cell lines and culture conditions: A549 cells and BJ-hTERT cell lines were used in this study. A549 is a cancer cell line derived from human lung adenocarcinoma, representative of the alveolar type II pneumocytes of the human lung [45,46,47,48]. This cell line was purchased from European Collection of Cell Cultures. BJ-hTERT cells are normal, non-tumor human fibroblasts immortalized by the transfection of the human telomerase reverse transcriptase gene. For routine maintenance, both cell lines were cultured in DMEM (Dulbecco’s Modified Eagle Medium) containing 4.5 mg/mL glucose and supplemented with 10% fetal calf serum (FBS), penicillin (50 U/mL), streptomycin (50 µg/mL), and L-glutamine (2 mM) at 37 °C in a humidified 5% CO_2_ atmosphere. Cells were subcultured when 90% of confluence was reached.

#### 2.1.1. Synthesis of N,N’-[1,3-phenylenebis(methylene))bis[N,N’-dimethyl-N-(1-hexadecyl)]-ammonium dibromide, 16-Ph-16

For the synthesis of the cationic p-16-Ph-16 gemini surfactant (Supplementary Materials Figure S1), α,α’-dichloro-p-xylene (11.9 g and 0.068 mmol) in dry acetonitrile was added dropwise to a stirred solution of *N*,*N*-dimethylhexadecylamine (40.1 g and 0.15 mmol) in 150 mL of acetonitrile. The mixture was refluxed for 96 h; the solvent was then removed under reduced pressure. A white solid was recrystallized from a (4/1) acetone/hexane mixture [49,50]. The critical micelle concentration (CMC) of the p-16-Ph-16 surfactant obtained by the surface tension was (8.0 ± 0.4) × 10^−6^ M. Upon cooling, a white solid was recovered by filtration. All products were recrystallized from ethyl acetate up to five times and dried under vacuum [50].

#### 2.1.2. Synthesis of Au@16-Ph-16 Precursor Gold Nanoparticles

For the gemini surfactant-functionalized gold nanoparticle preparation, 390 μL of an HAuCl_4_ 23 mM aqueous solution were added to 30 mL of a 16-Ph-16 gemini surfactant 4·10^−5^ M aqueous solution and stirred vigorously for 5 min in the absence of light. Subsequently, 100 μL of a freshly prepared 0.4 M NaBH_4_ aqueous solution was added drop by drop to the previously prepared yellow solutions, and the mixture was stirred moderately for 15 min in the darkness, acquiring a reddish color [34]. In this study, the CMC value of the surfactant was crucial for the parameter stability and optimization of nanosystem size; the relation between C_16-Ph-16_/CMC was equal to 5. As a result, aqueous solutions of Au@16-Ph-16 gold nanoparticles, with a concentration of 5.6·10^−8^ M, were obtained. Three Au@16-Ph-16 formulations (N_i_ in abbreviated form)—designated as N_1_, N_2_, and N_3_ for those prepared at different C_16-Ph-16_ concentrations of 1.94, 7.69, and 54.0 nM_,_ respectively—were explored in this study.

#### 2.1.3. Synthesis of Au@16-Ph-16/DNA–5-Fu Gold Nanoparticles

Once the synthesis of the precursor Au@16-Ph-16 was achieved, the 5-Fu transporter, Au@16-Ph-16/DNA–5-Fu, was synthesized starting from DNA/5-Fu complexes prepared under saturation, X = C_5-Fu_/C_DNA_ = 0.84. Method tuning also required the prior selection of the optimal relative Au@16-Ph-16 and DNA concentrations, R = C_Au@16-Ph-16_/C_DNA_, to ensure effective biopolymer compaction (see Section 3.1 for more details). Three formulations were explored at different C_5-Fu_, C_DNA_, and C_Au@16-Ph-16_ concentrations, working at fixed X and R ratios of 0.84 and 3.74·10^−4^, respectively. The distinct Au@16-Ph-16/DNA–5-Fu formulations, C_i_, were designated as C_1,_ C_2_, and C_3_. The concentration of the reactants was C_5-Fu_ = 0.25 μM, C_DNA_ = 0.30 μM, and C_Au@16-Ph-16_ = 0.011 nM for C_1_; C_5-Fu_ = 1.00 μM, C_DNA_ =1.19 μM, and C_Au@16-Ph-16_ = 0.045 nM for C_2_; and C_5-Fu_ = 7.5 μM, C_DNA_ = 8.9 μM, and C_Au@16-Ph-16_ = 0.33 nM for C_3_. To obtain stable Au@16-Ph-16/DNA–5-Fu complexes, the appropriate concentration of 5-Fu was added to an aqueous solution of ds-DNA at room temperature; the mixture was then gently stirred for 2 min. Subsequently, the Au@16-Ph-16 precursor was added to the DNA–5-Fu complex, and the mixture was stirred for 5 min. A change in the surface plasmon resonance (SPR) maximum wavelength (λ_MAX_) of the precursor from 520 to 527 nm was indicative of the stabilization of the formation of the nanocomplexes.

### 2.2. Methods

#### 2.2.1. (3-(4,5-. dimethylthiazol-2-yl)-2,5-diphenyltetrazole) (MTT) Viability Assay

Cells were seeded in 96-multiwell plates (611U96, Thermo Fisher Scientific Inc., Carlsbad, CA, USA) and treated after 24 h. The number of seeded cells was 3500 for A549 and 8000 for the BJ-hTERT (human fibroblasts immortalized by transfection of the Human Telomerase Reverse Transcriptase gene) cell line in each well. Cells were treated with different concentrations of C_i_ and N_i_ particles for 4 h, starting from solutions prepared at the 19× condition to obtain the final concentrations described in Section 2.1.2 and Section 2.1.3. After treatment was removed, and cells were kept in recovery for 48 h, and an MTT (thiazolyl blue tetrazolium bromide—A2231, Panreac Química S.L.U. Barcelona, Spain) assay was performed. In this assay, the reduction of MTT indicates cellular metabolic activity. Viable cells contain functional NAD(P)H (nicotinamide adenine dinucleotide phosphate, reduced form)-dependent oxidoreductase enzymes that reduced the MTT reagent to formazan, an insoluble crystalline product with a deep purple color. The MTT stock solution was prepared at a concentration of 5 mg/mL in phosphate-buffered saline (PBS) and kept at −20 °C. On the day of use, MTT was freshly diluted in DEMEM/HIGH GLUCOSE (Dulbecco’s Modified Eagle’s Medium/high glucose, D5796-500ML, Sigma-Aldrich-Merck KGaA, Darmstadt, Germany) (1:4). Then, 1 mL of the MTT working solution was added to each well, and incubation lasted 3 h. A lysis buffer (sodium dodecyl sulfate 10% SDS, 151-21-3 Sigma-Aldrich-Merck KGaA, Darmstadt, Germany; with 0.01 M hydrochloric acid HCl, 303112, Panreac Química S.L.U. Barcelona, Spain was then added to the cells, and plates were incubated overnight. Finally, the optical density of formazan was measured at 570 nm using the Multiskan Sky Microplate Spectrophotometer Labsystems Multiskan MS (Thermo Fisher Scientific Inc., Carlsbad, CA, USA).

#### 2.2.2. UV/Vis Spectroscopy

Absorbance spectra were measured using a CARY 500 SCAN UV−vis−NIR (Ultraviolet/Visible/Near Infrared) spectrophotometer (Varian, Markham, ON, Canada) at 298.2 K using a standard quartz cell with a 10 mm path length. The wavelength accuracy and the spectral bandwidth were ±0.3 and 1 nm, respectively. To ensure the complete formation of the nanocomplexes, the spectra of the Au@16-Ph-16 precursor and the compacted nanocomplex, Au@16-Ph-16/DNA–5-Fu, were recorded in the wavelength range of 800–400 nm. Moreover, the stability of the Ni and Ci nanoformulations was checked for at least two weeks, following the possible changes in UV–vis spectra from 200 to 800 nm over time (see Supplementary Materials Figure S2 and Table S1). Loading experiments were performed after the incubation of the prepared C_i_ nanocomplexes for 1 h and subsequent dialysis using a cellulose ester dialysis membrane for 24 h (Sigma-Aldrich-Merck KGaA, Darmstadt, Germany, MWCO = 10,000). Ci solutions were loaded into 15 cm segments of the seamless dialysis membrane, placed in 4 L beakers of water, and stirred slowly over the course of 24 h. The UV–vis spectra of the dialyzed samples were recorded in the wavelength range of 200–800 nm. The amount of 5-Fu loaded on the nanoparticles was assessed by means of decreasing the 5-Fu concentration from the initial solution and after correction from DNA concentration using UV–vis spectrophotometry (see Supplementary Materials Figure S2). Herein, drug loading (DL) was calculated as follows: %DL = (C_5-Fu_ concentration in Ci nanoparticles/C_5-Fu_ total concentration added) × 100 [51]. All solutions were measured at a fixed wavelength of 266 nm using the appropriate 5-Fu calibration curve (see Figure S3D).

#### 2.2.3. Fluorescence Spectroscopy

Fluorescence measurements were carried out at 298.0 K in a Hitachi F-2500 spectrofluorometer (Hitachi High Technologies America, Inc., Pleasanton, CA, USA) interfaced to a PC for reading and handling the spectra. To explore DNA/5-Fu interaction with Au@16-Ph-16 precursor gold nanoparticles, the concentrations of C_5-Fu_ = 41.7 μM and C_DNA_ = 50.0 μM were fixed, and the precursor concentration C_Au@16-Ph-16_ ranged from 0.187 to 51.6 nM. The excitation and the emission wavelengths were 266 and 340 nm, respectively. For the release assays, after 1 h of stabilization, the corresponding Ni nanoparticles needed to perform DNA decompaction and effective 5-Fu release were added to the initial solution of Ci nanocomplexes. The fluorescence of the Ci in the presence of the appropriate Ni systems (C_1_ and N_1_; C_2_ and N_2_; and C_3_ and N_3_) was stabilized in 15 min, at worst. The quantity of 5-Fu released from the C_i_ nanocomplexes (drug release: DR) was measured from the stabilized fluorescence spectra of the C_i_ and Ni in each case (see Supplementary Materials Figure S2), and it was calculated as follows: %DR = (C_5-Fu_ released upon N_i_ addition to Ci formulation/C_5-Fu_ total concentration added for Ci preparation) × 100 [51]. All solutions were measured at a fixed wavelength of 340 nm using the appropriate 5-Fu calibration curve (see Figure S4D). The drug half-life release (*t*^1/2^) of 5-Fu drugs upon the addition of Ni to Ci complexes was calculated from the plot of fluorescence intensity at 340 nm vs. time (see Supplementary Materials Figure S2).

#### 2.2.4. Circular Dichroism (CD) Spectroscopy

Electronic circular dichroism (CD) spectra were recorded with a BioLogic Mos-450 spectropolarimeter (Barcelona, Spain). A standard quartz cell with a 10 mm path length was used. The spectra were expressed in terms of molar ellipticity (θ). Scans were taken from 195 to 340 nm. For each spectrum, 5–10 runs were averaged at a constant temperature of 298.0 K with a 10 min equilibration before each scan. All the spectra were recorded at fixed biopolymer and 5-Fu concentrations of 50.0 and 41.7 μM, respectively, both in the absence and at increasing concentrations of C_Au@16-Ph-16_ from 0.187 to 18.7 nM.

#### 2.2.5. Atomic Force Microscopy Experiments

The AFM (Atomic Force Microscopy) images were obtained with a Molecular Imaging Picoscan 2500 (Agilent Technologies, Las Rozas, Madrid, Spain). For imaging in air, silicon cantilevers (Model Pointprobe, Nanoworld Neuchâtel, Switzerland) with a resonance frequency of approximately 240 kHz and a spring constant of 42 N/m were used. All AFM imaging was recorded in tapping mode, with scan speeds of about 0.5 Hz and data collection at 256 × 256 pixels. The acquired AFM images were flattened to remove the background slope [52]. The mica surface was modified and incubated for 20 min with a 0.1% (*v*/*v*) APTES solution. Subsequently, the surface was washed with ultrapure water and air-dried. Then, a total of 30 μL of isolated DNA (C_DNA_ = 0.3 μM), the 5-Fu/DNA complex (C_DNA_ = 0.3 μM and C_5-Fu_ = 0.25 μM), or the Au@16-Ph-16/DNA–5-Fu nanocomplexes (C_DNA_ = 0.3 μM and C_5-Fu_ = 0.25 μM) at different R ratios (R = 3.7 × 10^−6^–1.7 × 10^−4^) were dropped onto this modified surface. Note that the concentration of DNA was adjusted and diluted in such a way that the molecules were spread over the surface with no overlap. The adsorption of the prepared samples was for 30 min. Then, the sample was thoroughly rinsed with doubly distilled water and finally air-dried for AFM imaging.

#### 2.2.6. Dynamic Light Scattering (DLS) and Zeta Potential Measurements

The size and distribution of Au@16-Ph-16 and Au@16-Ph-16/DNA–5-Fu-compacted nanocomplexes were characterized by means of the dynamic light scattering (DLS) technique using a Zetasizer Model ZS-90 (Malvern, Worcestershire, UK). The samples were illuminated with a laser with a fixed detection arrangement of 90° to the center of the cell area, and the intensity fluctuation in the scattered light was then analyzed. At least 5 size measurements were taken for each sample, and the relative error for hydrodynamic diameter was calculated to be <5%. Results were obtained in terms of average hydrodynamic diameters, giving the percentage of different complexes obtained in solution. Zeta-potential (ζ) values were obtained to measure the electrophoretic mobility of the sample from the velocity of the particles using a laser Doppler velocimeter (LDV). A Zetasizer Nano ZS from Malvern Instrument Ltd. (Worcestershire, UK) was used. At least six zeta potential measurements were taken for each sample by using a DTS1060 polycarbonate capillary cell. The N_1_, N_2_, and N_3_ formulations prepared at the 19× concentration condition for the precursor and C_1,_ C_2_, and C_3_ described in Section 2.1.2 and Section 2.1.3 were explored using both techniques in the absence and in the presence of the PBS buffer (0.01×, ionic strength = 1.63 mM, and pH = 7.4) to study the effect of ionic strength on nanoparticle stability. Moreover, to check the stability of Ci formulations with ionic strength and concentration, measurements in the PBS buffer were also carried out at the 1× concentration condition.

#### 2.2.7. TEM and EDS Measurements

For TEM examinations of the size and morphology of isolated Au@16-Ph-16 gold nanoparticles and EDS analysis, a single drop (10 μL) of the aqueous solution was placed on a copper grid coated with a carbon film. The grid was left to air-dry for several hours at room temperature. TEM analysis was carried out with a high-resolution TEM-TALOS F200S electron microscope (FEI Company, Hillsboro, OR, USA) working at 200 kV. The resulting images were analyzed by using ImageJ 1.52a software, a free, public domain digital image processing program programmed in Java developed at the National Institutes of Health (1.52a, 2018, Bethesda, MD, USA). At least 200 particles were measured on the images in TIFF format, selecting the diameter of the bar with the corresponding tool and calibrating the bar with the corresponding units; the same tool was then used to measure the diameter of each nanoparticle, exporting the data obtained to an Excel spreadsheet (2016). From these measurements, the Au@16-Ph-16 particles were found to have a diameter of 5.6 ± 0.2 nm (Supplementary Materials Figure S2). EDS measurements were performed to verify whether or not the nanoparticles contained gold. These experiments were conducted using a TEM-TALOS F200S electron microscope (FEI Company, USA) equipped with an energy dispersive X-ray spectrometer and operating at 200 kV. Supplementary Materials Figure S2 shows EDS spectra in which K and L lines associated with the Au element can be seen, demonstrating the presence of gold on the nanoparticles. For the visualization of precursor and compacted nanosystems in cell samples, a Zeiss Libra electron microscope (Libra-120, Carl Zeiss Microscopy, Jena, Germany) working at 80 kV was used. In approximately 200 cells, the various concentrations of the free nanoparticle Au@16-pH-16 (N_1_, N_2_, and N_3_) and Au@16-Ph-16/DNA–5-Fu compact nanosystems (C_1_, C_2_, and C_3_), as well as their nanosystem controls alone, were visualized. The different cell groups were fixed in 1.6% glutaraldehyde (18426, Ted Pella, Inc., Redding, CA, USA) in cacodylate trihydrate solution (0.1 M and pH: 7.4) (12300, Electron Microscopy Sciences, Hatfield, PA, USA) for 1 h at room temperature and/or 277.0 K overnight and then placed in an automatic sample processor (EM.TP, Leica, Nussloch, Germany) with a 33 h and 25 minutes’ protocol, as follows. First, the samples were immersed in a sodium cacodylate trihydrate solution—0.1 M and pH = 7.4—for 5 min at 277.0 K for three times (X3). Samples were then post-fixed with a 1% osmium tetroxide solution (18453, Ted Pella, Inc., Redding, CA, USA) for 1 h at 277.0 K to preserve cell structure, and then they were washed in distilled water for 20 min at 277.0 K for three times (X3). Processing continued with contratinction with a 2% uranyl acetate solution to contrast the sample (22400, Electron Microscopy Sciences, Hatfield, PA, USA) for 2 h at 277.0 K. Next, the samples were dehydrated for subsequent inclusion in resins by washing in distilled water and acetone solution (10000, Electron Microscopy Sciences, Hatfield, PA, USA), increasing gradation from 50% to 90% for 30 min at 298.0 K and two immersions in 100% acetone for 20 min at 298.0 K. Inclusion began with immersion in a mixture of acetone and Spurr resin (14300, Electron Microscopy Sciences, Hatfield, PA, USA) at a 3:1 ratio for 1 h, followed by a mixture of acetone and Spurr resin at a 1:1 ratio for 2 h and another immersion in the same mixture at a 1:3 ratio for 2 h. The samples were then given three subsequent immersions in 100% Spurr resin lasting 2, 12, and 1 h, respectively, the inclusion being processed at 298.0 K for each step. Finally, they remained at 343.0 K for 7 h for the polymerization of the resins. Next, we proceeded to the re-cutting and semi-fine cuts with a glass blade (UC7 Ultramicrotome, Leica, Nussloch, Germany) in a standard range of 300 nm. To determine areas for ultra-fine cuts, we proceeded to mount sections on standard glass slides and used monochromatic staining with toluidine blue (251176.1606, Panreac Química S.L.U, Barcelona, Spain). Then ultra-fine cuts were made with a diamond blade that less than or equal to 70 nm. Finally, for sample visualization with a Zeiss Libra microscope, they were sectioned and mounted on 300 mesh copper grids. Images were obtained using the ITem software (Olympus, Tokyo, Japan).

#### 2.2.8. Confocal Microscope

Fluorescence was checked with a confocal laser scanning spectral optical microscope; a high speed, high resolution, and point-to-point laser scanning spectral unit; and the high speed confocal laser scanning online application (LSM7DUO, Carl Zeiss Microscopy, Jena, Germany). The software used for microscope control and image analysis was ZEN 2011. For a better visualization of the different cell bodies, the DAPI (nucleic acid staining of cells with 4′,6-Diamidino-2-phenylindole dihydrochloride) nuclei contratinction was carried out (Fluoroshield™ with DAPI, F6057, Sigma-Aldrich-Merck KGaA, Darmstadt, Germany). With two confocal channels for fluorescence, the microscope was equipped with highly sensitive detectors (QE (quantum efficiency) 70% or better) and a bright-field transmitted-light mode capability. There were individually variable confocal pinholes for each detection channel, and all lasers were of the maintenance-free diode or solid-state type without significant heat dissipation: a 405 nm laser diode at 50 mW, a 488 nm laser diode at 100 mW, a 532 nm diode-pumped solid-state laser at 75 mW, a 561 nm laser diode at 40 mW, and a 635 nm laser diode at 30 mW. Additional outlet from an existing LIVE Laser Module with polarization-preserving single-mode fiber; the splitting proportion between the outlets was freely variable via the software for the 405, 488, 532, and 561 nm laser lines.

## 3. Results and Discussion

### 3.1. Exploring Experimental Conditions to Obtain Optimal Au@16-Ph-16/DNA–5-Fu Vectors. Reversible Compaction of DNA/5-Fluorouracil Complexes

**Figure 1:**
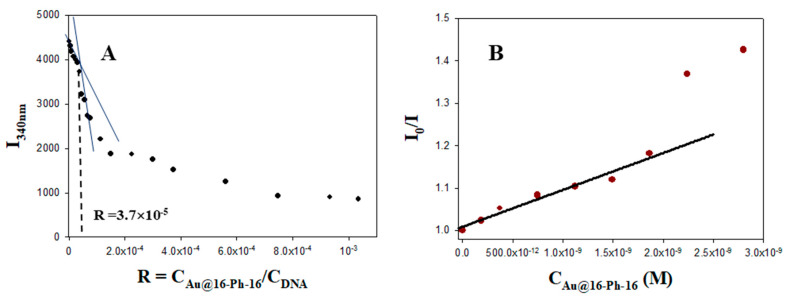
Fluorescence experiments with the DNA/5-Fu (5-fluorouracil) system in the presence of different C_Au@16-Ph-16_ at 298.0 K in a cacodylate buffer (ionic strength (I) = 1.63 mM and PH = 7.4); C_5-Fu_ = 41.7 μM and C_DNA_ = 50.0 μM. (**A**) Plot of I_340 nm_ versus the molar ratio, R = C_Au@16-Ph-16_/C_DNA_ (**B**) Stern–Volmer plot of DNA/5-Fu system at different C_Au@16-pH-16._

5-Fluorouracil is a chemotherapy drug that interferes with the growth of cancer cells. The alteration of the fluorescence emission spectra of the free and ds-DNA bound to 5-Fu is the same as that of ethidium bromide; that is, 5-Fu strongly emits due to its interaction with DNA bases [53], intercalation being its primary binding mode [54]. The extent of fluorescence quenching of DNA-bound 5-Fu in the presence of gold nanoparticles could be used to determine the extent of binding between the Au@16-pH-16 precursor and DNA molecules. Therefore, steady-state competitive binding experiments using Au@16-Ph-16 as a quencher at a fixed C_5-Fu_ and C_DNA_ concentrations may provide further information for the study of the binding of gold nanoparticles to ds-DNA and Au@16-Ph-16-DNA/5- fluorouracil for vector preparation. The emission spectra of 5-Fu alone and DNA/5-Fu in the absence and the presence of Au@16-Ph-16 are shown in Supplementary Materials Figure S2. As can be seen, an increase in the fluorescence emission was observed upon the addition of ds-DNA (red spectrum), while a quite evident decrease in emission intensity was observed upon the addition of different C_Au@16-Ph-16_ concentrations. The same behaviour can be observed in Figure 1 at the maximum emission wavelength of the 5-Fu probe. Two different tendencies in the fluoresence emission of the DNA/5-Fu system were operative as C_Au@16-Ph-16_ increased—the first corresponded to gold nanoparticles binding to the DNA/5-Fu complex and quenching; the subsequent pronounced decrease could be assigned to a displacement of the 5-Fu drug from its position between the bases of the polynucleotide to the aqueous solvent. The displacement of the 5-Fu probe may have been due to a conformational change induced by the gold nanoparticle precursor in the biopolymer, which, according to the results shown in Figure 1A, could have happened at R = 3.7 × 10^−5^ and C_Au@16-Ph-16_ = 1.87 nM.

**Figure 2:**
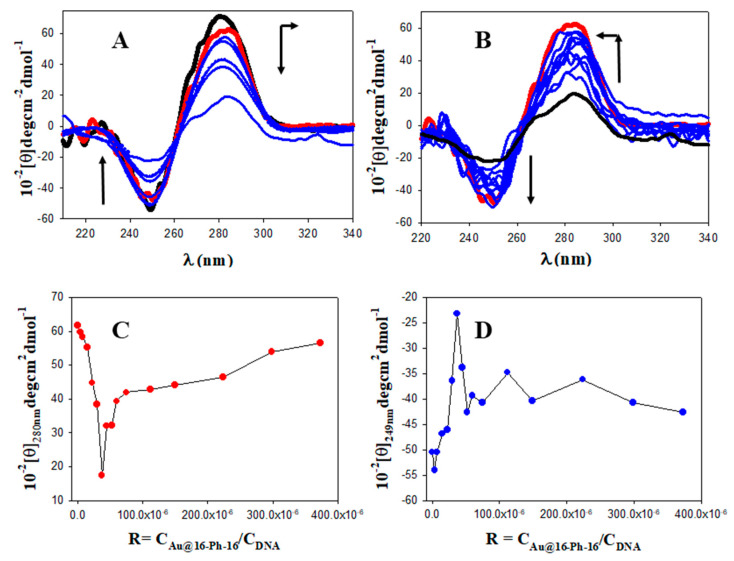
Circular dichroism (CD) titrations of the DNA–5-Fu system at different C_Au@16-Ph-16_ in a cacodylate buffer (I = 1.63 mM and PH = 7.4); C_5-Fu_ = 41.7 μM and C_DNA_=50.0 μM. The CD spectra of free DNA and the DNA/5-Fu complex in black and red, respectively. (**A**) First behaviour; arrow indicates change 0, 0.187, 0.373, 0.747, 1.49, and 1.87 nM of Au@16-Ph-16. (**B**) Second behaviour; arrow indicates change 1.87, 2.24, 2.61, 2.98, 3.73, 5.60, 7.47, 11.2, 14.9, and 18.7 nM of Au@16-Ph-16. (**C**,**D**) Plots of (θ)_280 nm_ and (θ)_249 nm_ (the maximum and minimum CD molar ellipticity) versus R, respectively. Dot symbols correspond to experimental data and solid lines to trend lines.

The quenching efficiency of gold nanoparticles was evaluated in Figure 1B using the Stern–Volmer constant (K_SV_):

$$\frac{I_{0}}{I}{=1+K}_{\mathrm{SV}}{\;\times \;C}_{\mathrm{Au}\text{@}16-\mathrm{Ph}-16}$$

where I_0_ and I are the fluorescence intensities in the absence and presence of Au@16-Ph-16, respectively. A K_SV_ value of (8.7 ± 0.7) × 10^7^ M^−1^ denotes a strong interaction of gold nanoparticles with the DNA/5-Fu complex. Note that deviation from linearity was observed in Figure 1B from C_Au@16-Ph-16_ = 1.87 mM, coincident with the R ratio at which the 5-Fu began its displacement to the solvent media. From the obtained results, it is evident that more information was needed to explain the complex fluorescent behaviour of the system and to explore the possibility of conformational changes as a function of C_16-Ph-16_. To this end, CD spectroscopy in conjunction with AFM constitutes a useful technique for diagnosing changes in DNA structure during ligand/DNA interactions. Figure 2A (in black) shows a CD spectrum characteristic of the right-handed B-form DNA form in the far UV region (220–320 nm), having a positive peak at 280 nm and a negative peak at 249 nm with approximately the same intensity. As we know, the structural alterations of the DNA backbone caused by its interaction with ligands are reflected in changes in this intrinsic CD spectrum. Since both 5-Fu and the Au@16-Ph-16 precursor had no CD signal in the region of 220–320 nm, the CD signal in the systems only could have been aroused by the DNA molecule. The observed bands in the CD spectrum of free DNA were caused by stacking interactions and the helical superstructure of the polynucleotide that provided an asymmetric environment for the bases [55]. When 5-Fu was added to the DNA system in the absence of gold nanoparticles (see Figure 2A in red) a moderate decrease in the intensity of the both bands was observed. This behaviour was a consequence of the intercalation of the drug into base-stacking that decreased the right-handedness of the DNA [50]. However, when Au@16-Ph-16 was added to the DNA/5-Fu complex, the decrease in both bands was greater and was coupled with a displacement of the positive band to a higher wavelength (Figure 2A), indicating DNA compaction, denaturation, and the unwinding of the double helix [56,57]. However, when the complete R ratios for the Au@16-Ph-16/DNA–5-Fu system were explored (see Figure 2B), the existence of a clear inflection point in the maximum and minimum CD molar ellipticity, (θ)_280 nm_ and (θ)_249 nm_ vs. the molar R ratio (R = C_Au@16-Ph-16_/C_DNA_ = 3.7 × 10^−5^; see Figure 2C,D), was indicative of the existence of two different conformational changes in the biomolecule. Note that these inflection points are presented at the same relative concentrations at which the deviation from linearity was observed in fluorescence spectroscopy. The second behaviour was opposite to the first and implied both an increase in the intensities of negative and positive CD bands and a displacement of the positive CD band to a lower wavelength, giving a final CD spectrum at C_16-Ph-16_ = 18.7 nM, which was very similar to the CD spectra of free DNA/5-Fu complex in solution (see Figure 2B in red). According to the observed CD trend, an increase in C_16-Ph-16_ above 1.87 nM (R = 3.7 × 10^−5^), the interaction of Au@16-Ph-16 gold nanoparticles with DNA/5-Fu complexes gave rise to larger DNA structures compared to the condensed ones.

On the basis of the foregoing discussion, it was clear that Au@16-Ph-16 induced reversible DNA compaction depending on the explored R range, where DNA condensation was favoured at low R ratios. However, both the morpholgy of the Au@16-Ph-16/DNA–5-Fu-compacted complexes and the nature of the larger structures detected by CD experiments at high R ratios required additional structural clarification. For this purpose, the highly sensitive AFM was crucial in distinguishing the different structures visualized at low and high R ratios, as it revealed some important information about the Au@16-Ph-16-DNA/5-Fu interactions. Supplementary Materials Figure S2 shows AFM topographic images of isolated double-stranded DNA adsorbed onto an APTES-modified mica surface in an extended-coil conformation.

**Figure 3:**
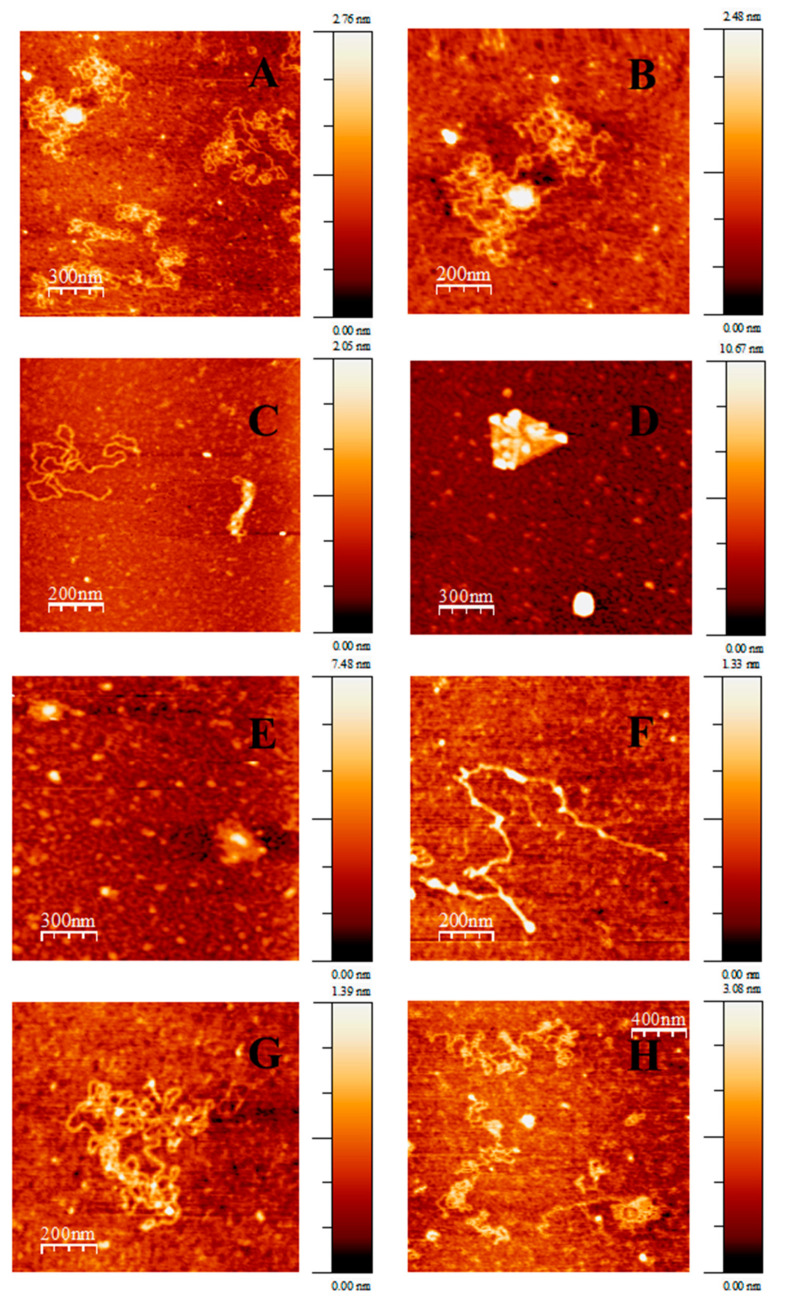
AFM topography images of DNA/5-Fu and C_i_ complexes adsorbed on 3-aminopropyltriethoxilane (APTES)-modified mica surface in a cacodylate buffer (I = 1.63 mM and pH = 7.4; C_DNA_ = 0.3 μM, C_5-FU_ = 0.25 μM) under different reaction conditions: (**A**,**B**) DNA/5-Fu complex, C_Au@16-Ph-16_ = 0 pM. (**C**) Intermediates of compaction; C_Au@16-Ph-16_ = 1.10 pM. (**D**,**E**) Ci complexes at a compact stage; C_Au@16-Ph-16_ = 11.1 pM. (**F**,**G**) Intermediates of decompaction; C_Au@16-Ph-16_ = 34.0 pM. (**H**) Ci complexes at the decompacted stage; C_Au@16-Ph-16_ = 50.0 pM.

In contrast, Figure 3A,B shows the structural effect of 5-Fu on DNA structure. As can be seen from the figure that the partial intercalation of 5-Fu on the DNA bases induced moderate changes in the secondary DNA structure that were compatible with partial DNA condensation. Polynucleotydes forming independent loops, as well as a few globules, were observed. This experiment was carried out in the absence of gold nanoparticles. However, when Au@16-Ph-16 gold nanoparticles were added to the DNA–5-Fu complex at low C_Au@16-Ph-16_, a compaction process from an elongated coil to a compact globule was observed. In particular, at a molar ratio of R = 3.7 × 10^−6^, Figure 3D shows the formation of different polymer aggregates and DNA loops, with the formation of some crossover points, that constituted intermediates of the condensation process [32]. With the further addition of Au@16-Ph-16 to the DNA/5-Fu complex, the molar ratio was R = 3.7 × 10^−5^; only compact globule structures appeared, indicating the completion of the compaction process (Figure 3D,E). Note that when the number of gemini surfactants per DNA/5-Fu complexes in the solution increased as the C_Au@16-Ph-16_ did the same, the negative charges in the DNA chain were shielded by the surfactant bound to the surface of the polymer [33]. This special situation favored intramolecular DNA interactions and DNA–DNA interconnections between distinct DNA helices, inducing the collapse of DNA/5-Fu structures around nucleation centers [33,58]—DNA strands wrapped around the surfactant aggregates, shielding the charge of the system and forming the compacted Au@16-Ph-16/DNA–5-Fu nanocomplexes. Once the compaction process was completed, the further addition of Au@16-Ph-16 entailed an increment in the size of the complexes (see Figure 3F,G). This behavior indicated that a strictly different conformational change began to be observed, which was consistent with the CD results. The presence of long, thick, flattened DNA structures (Figure 3F) in conjuction with flower-like structures (Figure 3G) indicated that a decondensation event began at R = 1.13 × 10^−4^. Finally, the decondensation process was complete at an R = 1.67 × 10^−4^ molar ratio, i.e., in the region of the CD titration curve in which the characteristics of the CD spectra of DNA/5-Fu complexes returned to those corresponding to the extended B-DNA form. As can be seen in Figure 3H, the DNA/5-Fu complexes became a structure similar to the free DNA in solution, in which extended DNA chains could be seen on the mica surface.

Hence, according to the results obtained from CD, AFM, and fluorescence spectroscopy measurements, three important conclusions could be drawn: (i) compacted Au@16-Ph-16/DNA–5-Fu nanocomplexes were formed at low R = 3.7 × 10^−5^, (ii) the compaction process was reversible when increasing concentrations of the Au@16-Ph-16 precursor were added, and (iii) the decompaction of the nanosystem was accompanied by the release of the 5-Fu drug to the solvent media. The last outcome is worthy of note since it delineated a fresh strategy to induce drug release from the compacted nanosystem after internalization.

### 3.2. Physicochemical Characterization and Stability of Au@16-Ph-16 and Au@16-Ph-16/DNA–5-Fu Nanosystems

**Figure 4:**
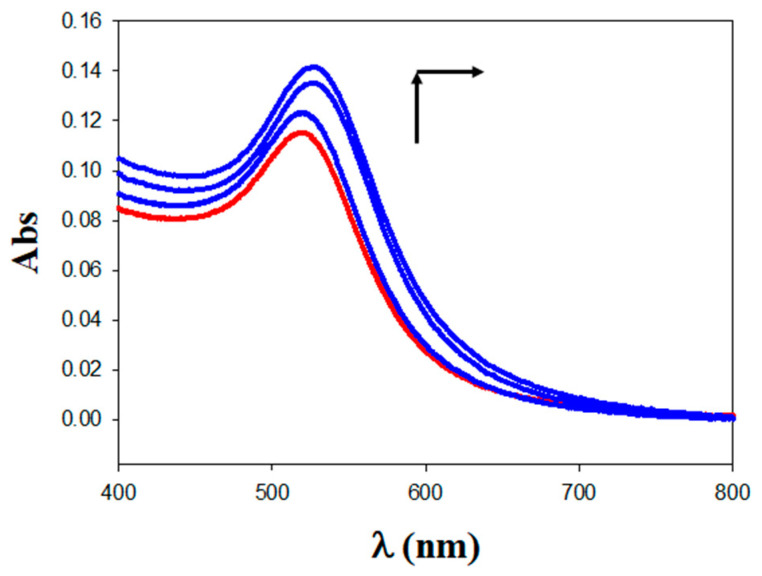
Absorbance spectra of nanocomplexes under different reaction conditions in a cacodylate buffer (I = 1.63 mM and pH = 7.4); C_Au@16-Ph-16_ = 5.6 mM in all the spectra. C_DNA_ = 0 M (in red; Au@16-Ph-16), X = C_5-Fu_/C_DNA_ = 0.84, and C_DNA_ = 1.0, 10.0, and 100.0 μM arrow change (in blue; Au@16-Ph-16/DNA–5-Fu).

Figure 4 shows the UV–visible plot for the free Au@16-Ph-16 precursor nanoparticle (in red), where the position of the plasmon peak is λ_spr_ = 520 nm. According to the H. Wolfgang correlation, λ_spr_ = 512 + 6.53 × exp (0.0216 × d), where d is the diameter of the gold core; this value of λ_spr_ approximately corresponds to a 9 nm core size AuNPs [59]. It is important to note that as C_DNA_ was increased from 1.0 to 100 μM in Au@16-Ph-16/DNA–5-Fu preparations, a displacement in the position of the SPR maximum to a higher wavelength, together with an increase in the absorbance intensities, was registered with reference to the precursor (Figure 4 in blue). The observed increase in the λ_spr_ maximum position from 520 to 527 nm and intensities, without a significant broadening of the SPR band, denoted the effective formation of the Au@16-Ph-16/DNA–5-Fu complexes [60]. Furthermore, the Wolfgang correlation provided a rough estimation of the diameter of the DNA/5-Fu-coated nanoparticles of about 39 nm. Additionally, the observed increase in λ_spr_ intensity when we compared the UV–vis spectrum of the free Au@16-Ph-16 precursor with the functionalized DNA–5-Fu nanoparticles could be related to the nature and morphology of the compacted derivate. According to Agyapong et. al., the production of more regular and better dispersed functionalized nanoparticles with respect to the precursor creates a new system with high sensitivity that is responsible for such a hyperchromic effect [61].

Furthermore, the stability of the Ni and Ci nanoformulations was checked by studying the possible modifications of the complete UV–vis spectra from 200 to 800 nm over time after 1 h of preparation and then, successively, 48 h, 72 h, 5 days, 1 week, and 2 weeks. Supplementary Materials Figure S2 and data from Table S1 show that the absorbance curves practically superimposed, and both the position of the plasmon’s maximum surface and band form were maintained over time for at least two weeks. Thus, the stabilization and non-aggregation of the complexes was verified from these results. On the other hand, the percentages of drug loading and release were also evaluated for the formulations studied in this work (see Figures S3–S5). In fact, insufficient drug loading with uncontrolled drug release represents one of the key challenges to be overcome for the possible clinical translation of the developed nanomedicine [51]. Drug loading and release parameters were spectrophotometrically calculated (see Section 2.2.2 and Section 2.2.3 for more details), and the obtained results are given in Table 1. These results clearly showed that a significantly larger amount of 5-Fu was loaded on Ci compacted nanosystems. This was due, on one hand, to the high affinity for the DNA biomolecule of the 16-Ph-16 cationic gemini surfactant on the Au@16-Ph-16 nanoparticle surface [33] and, on the other hand, to the strong intercalation of 5-Fu between the DNA bases in the carrier. Furthermore, as can be plainly observed, a high percentage of 5-Fu was released from the Ci-compacted nanocomplexes after the appropriate Ni nanoparticle addition in only 15 min of stabilization, at worst. These results showed that the release of the 5-Fu drug from the Ci formulations could be controlled by the careful tuning of the DNA conformation after precursor addition, indicating the goodness of the method.

Regarding the zeta potential and DLS size distribution of different formulations, Table 2 shows that the positive zeta potential values of the Au@16-Ph-16 precursors (N_i_) and compacted Au@16-Ph-16/DNA–5-Fu complexes (C_i_) varied between +30 and +40 mV and between −29 and −33 mV in water, respectively. Note that the observed zeta potential values, together with the single zeta potential peak presented for each formulation (Supplementary Materials Figures S10 and S11), indicated that all the systems are stable from flocculation and aggregation. Thus, a zeta potential value of around +/−30 mV or higher was considered optimum to attain better physical colloidal stability [62]. Moreover, the positive zeta potential values of the Au@16-Ph-16 precursors were compatible with cationic gemini surfactant functionalization. On the other hand, DNA in water has a highly negative zeta potential due to the negative charge conferred by the phosphate groups on its backbone [63]. Therefore, the interaction of cationic Au@16-Ph-16 nanoparticles with a highly negatively-charged DNA/5-Fu complex resulted in Au@16-Ph-16/DNA–5-Fu with a negative zeta potential.

In addition, Table 2 shows the hydrodynamic diameter of the Ni and Ci formulations in water solution. Again, the size distribution by number of different nanocomplexes in Supplementary Materials Figures S12 and S13 shows the existence of a single peak, revealing the formation of monodispersed and stable nanosystems. Furthermore, the size of the Ci nanosystem is worthy of note; that is, the size distribution of extended DNA biopolymer in the water solution was about 1000 nm [32]. Thus, the great decrease in the diameter corresponding to Au@16-Ph-16/DNA–5-Fu nanocomplexes, which varied from 16 to 88 nm, in comparison with the diameter of the free DNA in solution, agreed with the biopolymer compaction process induced by the precursor described in Section 3.1. Note that the great decrease in the diameter observed for the C_3_ formulation in comparison with C_1_ and C_2_ could be attributed to the higher C_DNA_ concentration used for the C_3_ coating, which led to additional stability [64]. Thus, in accordance with absorbance experiments in Figure 4, an excess of DNA led to stable solutions, resulting in shifts in the SPR peak and significantly negative zeta potentials. Regarding the effect of low ionic strength on the charge and stability of gold nanosystems, Table 2 and Supplementary Materials Figures S14 and S15 reveal a decrease in zeta potential, which was accompanied by a moderate increase in the diameter size of the N_i_ precursor nanoparticles with respect to the water media. From this result, it was evident that electrostatic attraction forces between the cationic Au@16-Ph-16 nanoparticles and anionic counter-ions drove the aggregation phenomena. Furthermore, since a low zeta potential value could result in particle aggregation due to the van der Waals attractive forces acting upon them [62], the self-assembly effect of Au@16-Ph-16 in the presence of salt was more pronounced for the N_1_ and N_2_ formulations that presented low zeta potential charges. This means that in the present study, the higher the C_Au@16-Ph-16_ concentration in the N_i_ formulation, the more stable the obtained nanoparticles were for possible medical applications. However, the effect of low ionic strength on the charge and stability of the C_i_ compact nanosystem’s stabilization was starkly different.

In this sense, Table 2 and Figures S14–S17 reveal an increase in the charge for different Ci formulations with respect to water as dispersal took place. However, the size values obtained for the Ci nanosystems in water and PBS at the 19× condition were very similar considering the standard deviation. Moreover, through the moderate aggregation phenomena observed for Ni precursors at a low salt content, long ds-DNA polymers could protect Au@16-Ph-16 against salt-induced aggregation; this protective effect was more pronounced at higher biopolymer concentrations (formulation C_3_). As we know, a low salt addition to DNA can cause conformational changes in the biomolecule and can also alter its interaction with gold nanoparticles [56,57,64]. The observed behavior is compatible with the DNA shrinkage induced at low salt content that contributes to nanoparticle stabilization [64]. As a result, the Ci nanosystems synthesized from the precursor were highly charged, compacted, and stabilized at low salt content. This finding was crucial to guarantee their use for in vivo delivery of drugs and to ensure efficacy in nanomedicine design. However, when we explored Ci and Ni formulations at the 1X condition, the effect of low salt was more evident in the compacted nanocomplexes, excluding the C_3_ formulation (see Supplementary Materials Figures S18 and S19). In particular, the size values of the compacted C_1_ and C_2_ nanocomplexes were higher than 100 nm, in agreement with AFM results in Figure 3C,D. The observed behavior in which low concentrated nanosystems were more affected by salt is consistent with the Debye screening effect. Thus, screening length moderately increased with the low salt content, decreasing the repulsive electrostatic interactions among nanoparticles and favoring nanoparticle aggregation [65]. Therefore, we theorize that the most stable and adequate formulations are those with high C_Au@16-Ph-16_ and C_DNA_ (N_3_ for Au@16-Ph-16 and C_3_ for Au@16-Ph-16/DNA–5-Fu-compacted nanosystems), which could be the most appropriate synthesis for cancer treatment applications.

### 3.3. In Vitro Biocompatibility of Gold Nanosystems

**Figure 5:**
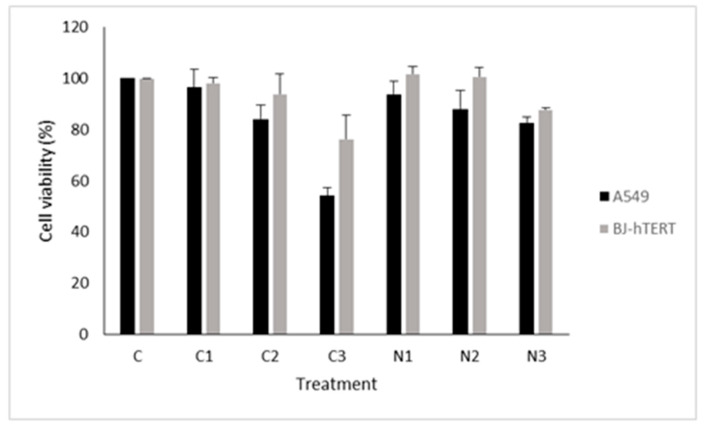
Cell viability for Ci and Ni formulations in A549 cancer cells and healthy BJ-hTERT cells. C is designated as the control, and it corresponds to cells without treatment.

In order to check the biocompatibility and selectivity of Ni and Ci formulations against lung cancer cells, MTT assays were carried out on both A549 cancer cells and BJ-hTERT healthy cells. Cytotoxicity was assessed 48 h after treatment (see Figure 5).

The results showed that Ci treatment induced higher cytotoxicity in A549 than in BJ-hTERT after 48 h of recovery time. A clear decrease in cell viability could be seen for the higher C_3_ concentration. Note that this finding was in good agreement with DLS and zeta potential results, according to which the most stable, highly charged, and smallest-sized formulation was C_3_. The Ni nanosystem caused a slight increase in cytotoxicity in the cancer cell line as compared with the primary cells. Furthermore, we found a lower viability for N_3_ than for N_1_ and N_2_. Again, this result was in good agreement with the physicochemical properties of the N_3_ nanosystem. As previously described, the highest positively-charged and smallest-sized Ni formulation was N_3_. We theorize that these characteristics provided the nanoparticle with a high stability, furnishing its interaction with cells.

### 3.4. Internalization of Au@16-Ph-16 and Au@16-Ph-16/DNA–5-Fu Nanosystems

**Figure 6:**
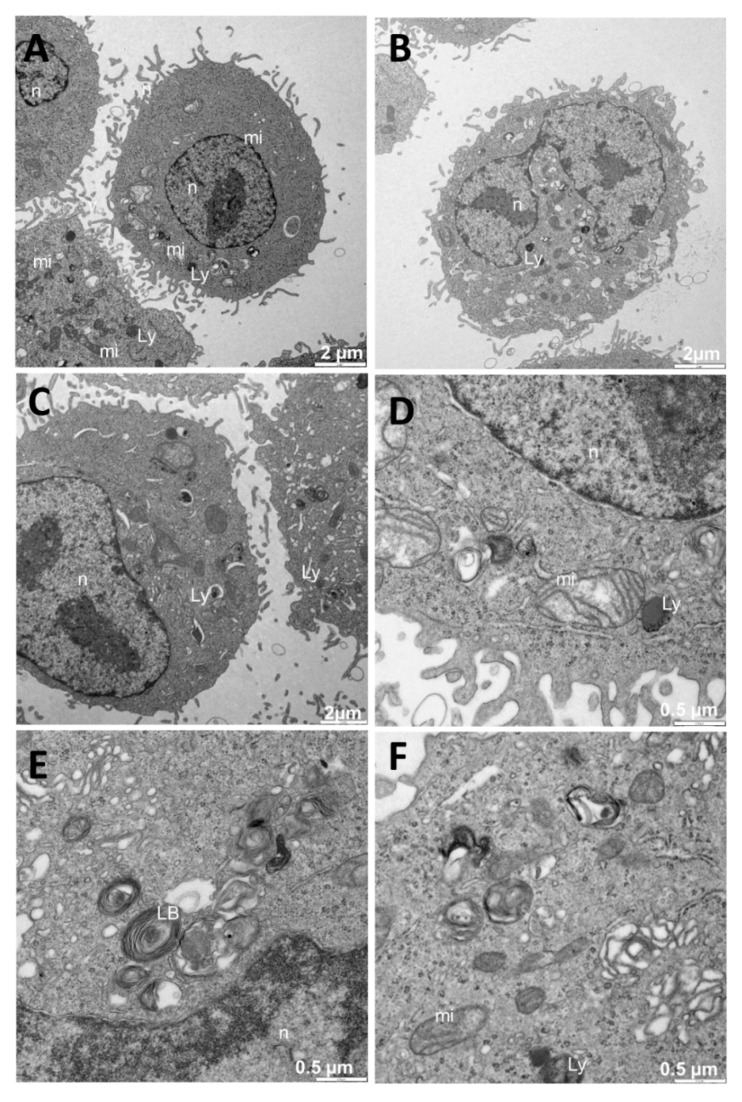
TEM images of A549 cells without any treatment (negative control): (**A**–**F**) with the typical morphology of the cell type, including the presence of lamellar bodies (**E**). Abbreviations: LBs: lamellar bodies; Ly: lysosome; mi: mitochondria; n: nucleus.

To gain insight into the behavior and effectiveness of Ci and Ni nanoparticles in cancer and normal cells, the cellular uptake of different nanosystems was studied. Below, we describe the results of the experiments carried out with TEM and confocal microscopy exploring the internalization of the nanosystem. First, we carried out experiments with the different C_i_ and N_i_ formulations using TEM. This microscopy technique permitted the visualization of the organelles, and it could serve to verify the presence or absence of nanoparticles inside the cells. In the study carried out on A549 cells without any treatment, we observed that the cells had organelles typical of the type to which they belonged (see Figure 6). Some vesicles were visualized, though without dense bodies inside. Since A549 cells were compatible with those of type II pneumocytes, they showed the presence of lamellar bodies (Figure 6E), a fundamental component in the synthesis of lung surfactants. Numerous mitochondria (mi) were observed to be quivering. Moreover, vesicles with dense content compatible with lysosomes (Ly) were present (Figure 6A–F). There were no dense bodies outside either, and the appearance of the cells was completely normal considering their typology; therefore, it corresponded to a negative control.

**Figure 7:**
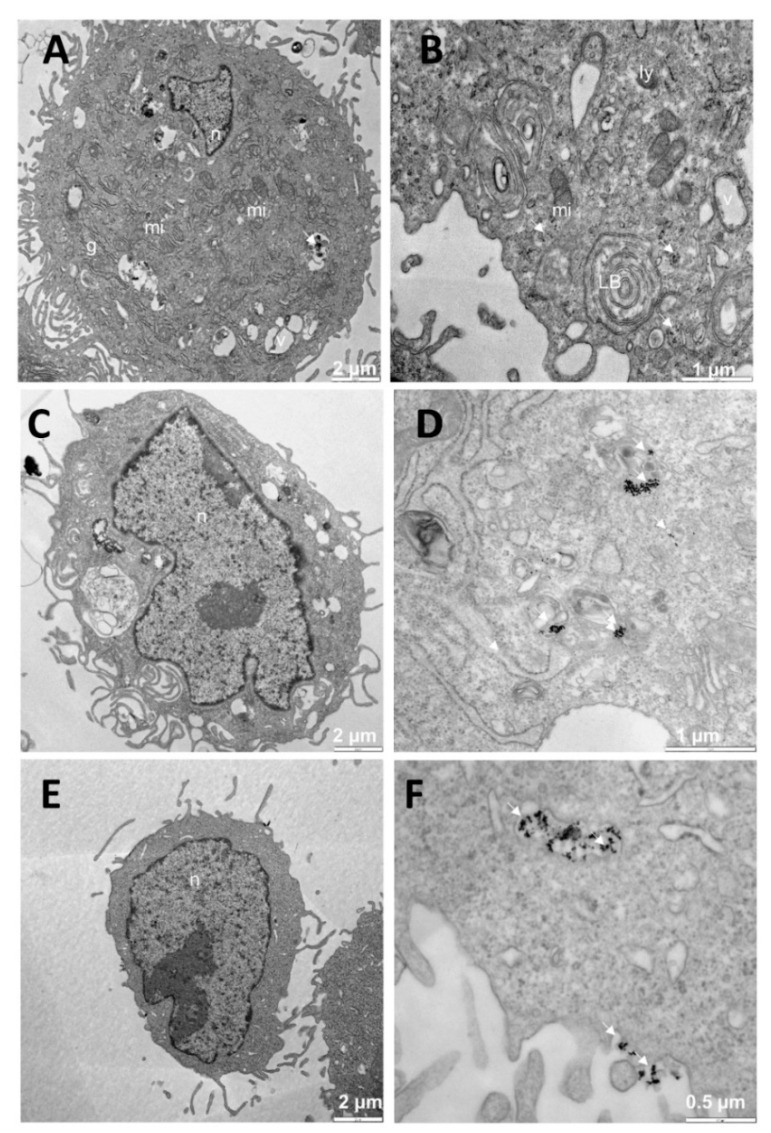
TEM images of A549 cells treated with Au@16-pH-16/N_1_ (**A**,**B**), N_2_ (**C**,**D**), and N_3_ (**E**,**F**). The dense bodies indicated by an arrow were compatible with the gold core of the nanoparticles. Abbreviations: LBs: lamellar bodies; Ly: lysosome; mi: mitochondria; n: nucleus.

With regard to the treatment with free N_i_ nanoparticles at different C_Au@16-Ph-16_, the images clearly show the presence of Au@16-pH-16 gold nanoparticles (Figure 7A,B,D,F). To ensure the presence of dense particles compatible with gold nanoparticles and to effectively distinguish them from other particles, low contrast photos in which small elements such as ribosomes were blurred but the dense particles remained clearly visible, were taken. In cells treated with the N_1_ precursor, dense bodies are shown in vesicles (v) (Figure 7A,B). In addition, vacuoles, and lamellar bodies (LBs) can be observed.

The sampling showed cells at different stages, including totally normal cells. Furthermore, for A549 cells treated with the N_2_ formulation (Figure 7C,D), the observed morphology was similar to those treated with N_1_ (Figure 7A,B): very well-preserved, with intact nuclei and showing dense bodies compatible with nanoparticles. Finally, in A549 cells treated with N_3_ (Figure 7E,F), dense bodies that were compatible with nanoparticles were observed both inside and outside the cell.

**Figure 8:**
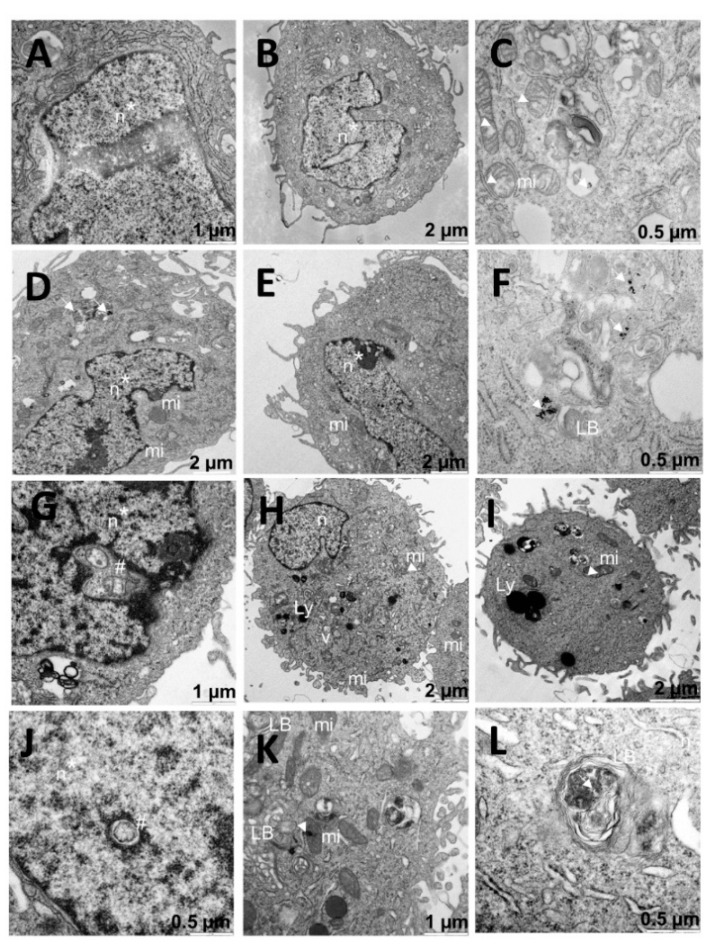
TEM images of A549 cells treated with C_1_ (**A**–**C**), C_2_ (**D**–**G**), and C_3_ (**H**–**L**). Arrows indicate dense bodies compatible with the gold cores (**D**,**F**). Arrowheads indicate possible damage to mitochondria with small vacuoles or dense internal clusters (**C**,**I**,**K**). Asterisks indicate damaged nuclei, and hashmarks indicate structures invading nuclei (**A**,**B**,**D**,**E**,**G**,**I**). Abbreviations: LBs: lamellar bodies; Ly: lysosome; mi: mitochondria; n: nucleus; v: vesicle.

In contrast, tests carried out with the different C_i_ formulations of the Au@16-Ph-16/DNA–5-Fu-compacted nanosystem on A549 cells showed a morphology of the nucleus that indicated the involvement and possible initiation of its degradation (Figure 8A,B,D,E,G,J). In addition, such structures were shown in the nuclear interior (Figure 8G,J). Note also that these structures were surrounded by a membrane, which made their location within the nucleus extremely abnormal. Moreover, dense bodies that were compatible with the gold core of nanoparticles were identified and distributed throughout the cells (Figure 8C,D,F,K,L) and were also present in organelles such as the mitochondria (Figure 8K). The morphology of the vacuoles was reminiscent of that of mitochondria. In fact, there were mitochondria that presented some fenestrations (Figure 8C,I). We deduced that the nanosystem would probably have had an implication in the destruction of some organelles and/or that nuclear involvement initiated the process of cellular degradation. Interestingly, many of the cells treated with C_2_ had an apparently normal nucleus, although nuclear damage was also found (Figure 8G). On the other hand, affected nuclei were found in most of the tested cells. Numerous vesicles compatible with lysosomes (Figure 8H,I,K,L) were found throughout the cells. As is known, understanding the endocytosis process of AuNPs is important for drug delivery and photodynamic therapy applications [66]. Additionally, in previous studies, the endocytosis of AuNPs was found to be dependent on not only the surface coating but also particle size [67,68,69]. In principle, in the present study, the used nanosystems not only entered the cell by endocytosis but were also internalized in the nucleus. Moreover, the large number of vesicles and lysosomes may have indicated that the drug acted on the cell by freeing itself from the nanosystem. This action was accompanied by the removal of gold from the cells by the inclusion in vesicles, the subsequent expulsion of the vesicular contents by means of the fusion of the vesicular membrane with the cytoplasm, and the expulsion of the vesicular contents to the outside.

**Figure 9:**
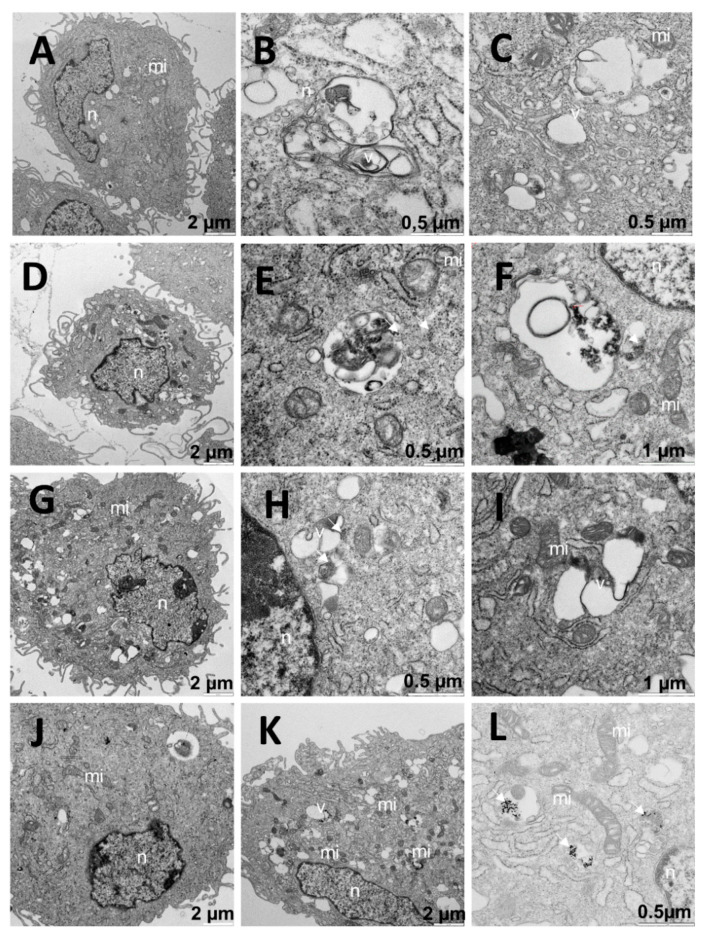
TEM images of BJ-hTERT cells and control cells (**A**–**C**); TEM images of cells treated with Au@16-pH-16, N_1_ (**D**–**F**), N_2_ (**G**–**I**), and N_3_ (**J**–**L**). Dense bodies compatible with gold cores of nanoparticles are indicated by arrows. Abbreviations: mi: mitochondria; n: nucleus; v: vesicle.

**Figure 10:**
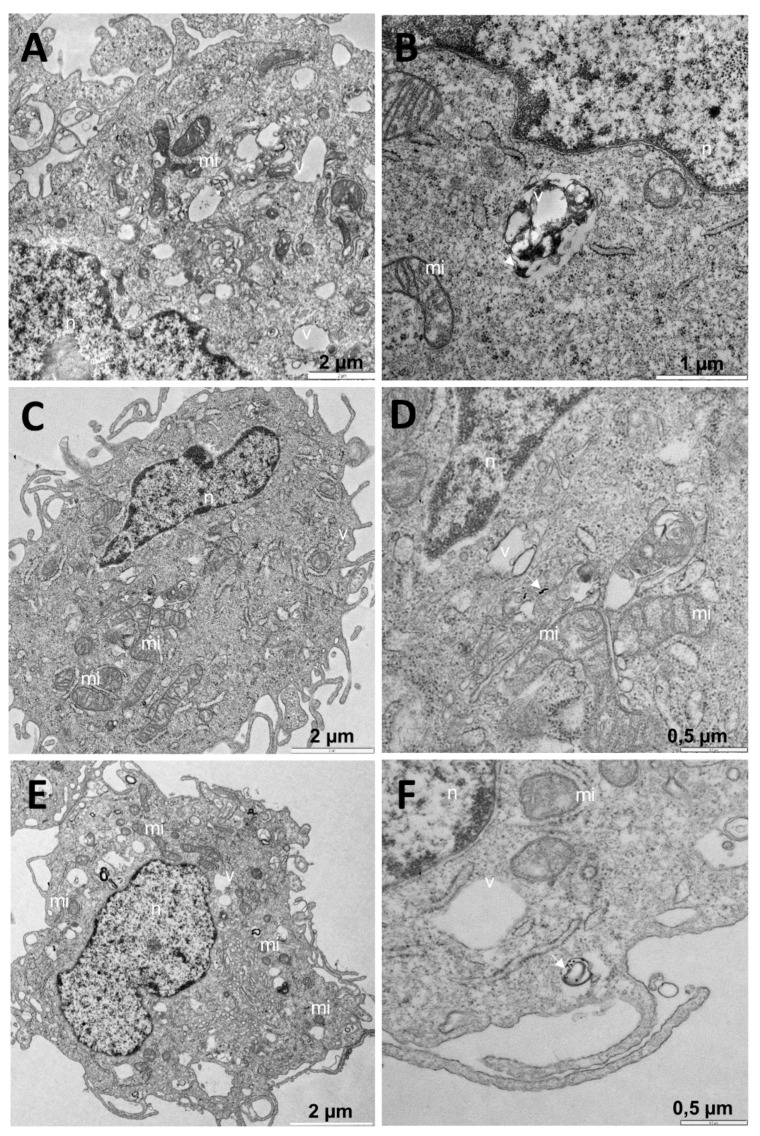
TEM images for BJ-hTERT cells, treated with Au@16-Ph-16/DNA–5-Fu-compacted nanosystems: C_1_ (**A**,**B**), C_2_ (**C**,**D**), and C_3_ (**E**,**F**), arrows indicate the presence of nanoparticles (**D**,**F**). Abbreviations: mi: mitochondria; n: nucleus; v: vesicle.

In the experiments carried out with non-tumor BJ-hTERT cells, we followed the same procedure. First, we studied the cells without any treatment (Figure 9A–C), and cells with Au @16-pH-16 at different C_Au@16-Ph-16_ were subsequently visualized (N_1_: Figure 9D–F; N_2_: Figure 9G–I; and N_3_: Figure 9J–L). Finally, the effects of the addition of the Au@16-Ph-16/DNA–5-Fu-compacted nanosystem on cells at different C_i_ formulations were explored (Figure 10).

In general, cells without treatment (Figure 9A–C) presented a normal appearance compatible with the tested cell type. In the treatments carried out with N_2_ and N_3_, a large number of vacuoles were observed in many cells (Figure 9G,K). Dispersed throughout the cytoplasm and enclosed in vesicles, dense bodies compatible with the dense cores of the nanoparticles were observed (Figure 9F,H,L). Note that in this case, the photographs were not contrasted to avoid confusion with dense organelles such as ribosomes. In cells treated with N_1_, dense bodies encompassed in vesicles compatible with nanoparticles could be observed that were arranged in an aggregate form in large vesicles (Figure 9E,F), resulting in two mean sizes of 5.1 ± 1.3 and 30 ± 7 nanometers, denoting that that N_1_ nanoparticles aggregated in part inside the cell. Thus, these formulations that were stable in original water solutions may have aggregated upon exposure to biological media due to the presence of aggregation-inducing molecules/species (such as salt), which may have significantly altered the uptake extent, rate, and mechanism [70]. Moreover, the appearance of the mitochondria was normal, although a greater number were observed in most cells (Figure 9D,G,K) than in the controls (Figure 9A).

In the tests carried out with different formulations of the Au@16-Ph-16/DNA–5-Fu-compacted nanosystem on BJ-hTERT cells, most of the cells presented a nucleus with normal morphology (Figure 10A–F). In some cells, dense bodies were observed on the surface, encompassed in vesicles that were compatible with nanoparticles, but it was not possible to distinguish whether they were entering or leaving (Figure 10F). Such structures were also dispersed throughout the cell cytoplasm without being contained in vesicles (Figure 10D). In all the treatments, the appearance of the mitochondria was normal (Figure 10A–F). However, in most of the cells, the number of mitochondria was much higher compared to the untreated cells (Figure 10C,E), which also occurred in cells treated with the Ni-free nanoparticle (Figure 10D,G,K).

**Figure 11:**
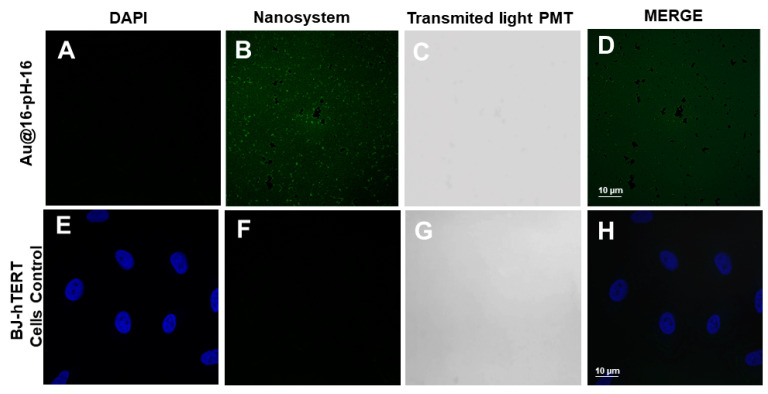
Confocal photomicrographs. (**A**–**D**) Au@16-pH-16 directly on the cell-free slide. (**E**,**F**) BJ-hTERT cells labeled with DAPI and without any treatment. (**A**–**E**) The presence or absence of N_3_ nanoparticles with fluorescence (**B**–**F**) and transmitted light (**C**–**G**). (**D**–**H**) show the result of the merge.

Therefore, in light of the obtained results exploring TEM images of Ci and Ni samples, it seems clear that the internalization of N_3_ and C_3_ formulations induced a better cell degradation in lung cancer cells compared to the other samples. As is known, smaller and spherical nanoparticles generally seem to more efficiently enter and exit the cell [71]. Therefore, the high degree of internalization obtained for smaller N_3_ and C_3_ nanosystems was in line with previous investigations of the cellular uptake of distinct nanoparticles. Moreover, such compositions did not only present a greater effect on the decrease in viability for cancer cells vs. healthy cells; they also highlighted those characteristics that made them more effective and stable nanosystems for possible medical applications via their high charge (either positive or negative) and small-sized distribution. It is also noteworthy that smaller nanoparticles seemed to more efficiently enter and exit the cell. Therefore, taking the special characteristics of N_3_ and C_3_ formulations into account in an attempt to assess internalization, confocal microscope studies were performed on the BJ-hTERT cells. Nanoparticles on slides and cells without any treatment were used as controls (Figure 11). 

**Figure 12:**
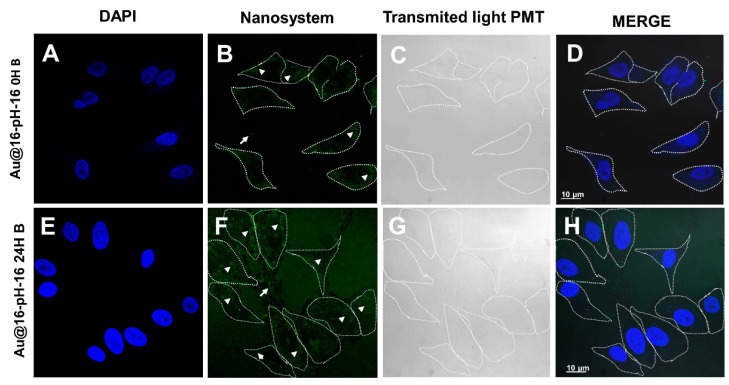
Confocal photomicrographs showing BJ-hTERT cells treated with free nanoparticle N_3_ Au@16-pH-16 at 0 h (**A**–**D**) and 24 h after treatment (**E**–**H**). (**A**,**E**,**D**,**H**) the cell nuclei labeled with fluorochrome DAPI. The presence of nanoparticles with fluorescence is shown in (**B**,**F**) and with transmitted light in (**C**,**G**). (**D**–**H**) show the result of the merge. Dashed lines mark the cell contours. Arrows indicate nanoparticles on the cell exterior, and arrow heads show nanoparticles internalized in cells.

**Figure 13:**
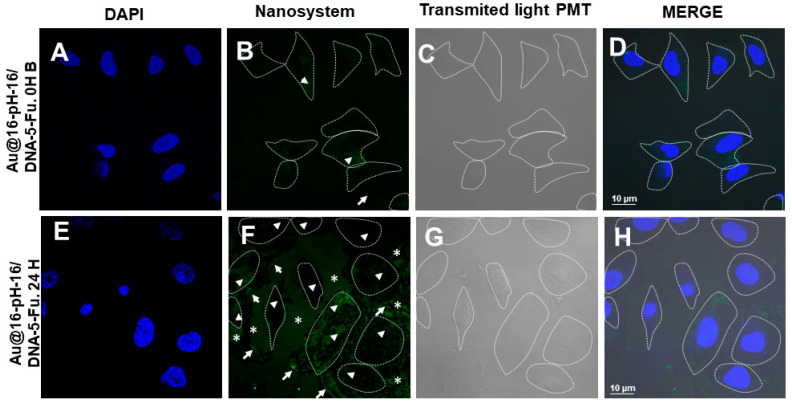
Confocal photomicrographs showing BJ-hTERT cells treated with C_3_ Au@16-Ph-16/DNA–5-Fu-compacted nanosystems at 0 h (**A**–**D**) and 24 h after treatment (**E**–**H**). (**A**,**E**) show the cell nuclei labeled with fluorochrome DAPI. The presence of nanoparticles with fluorescence is shown in (**B**,**F**) and with transmitted light in (**C**,**G**). (**D**–**H**) show the result of the merge. Dashed lines mark the cell contours. Arrows indicate nanoparticles on the cell exterior, and arrow heads show nanoparticles internalized in cells. Asterisks indicate possible cytoplasmic remains.

The administered treatments were Au@16-pH-16 (Figure 12) and Au@16-Ph-16/DNA–5-Fu (Figure 13) at 0 and 24 h. In the positive control (Figure 11A–D), the dispersed distribution of the nanoparticles was observed throughout the slide. As a negative control, the cells were not treated with any nanosystem (Figure 11E–G); only a DAPI fluorochrome counterstain specific for cell nuclei was used. As shown in the figures, no structures that were compatible with nanoparticles were observed; only cell nuclei stained with DAPI were evident (Figure 11E,H).

In the treatment of BJ-hTERT cells with Au@16-pH-16 (Figure 12), nanoparticles were observed in the cells at 0 h in small amounts (Figure 12A–D), becoming more evident at 24 h after performing treatment (Figure 12E–H). It seems that the nanoparticles may have been rapidly internalized in some cells. Structures compatible with nanosystems were also observed outside the cell, again more evident at 24 h (Figure 12B,F). To establish a control over the internalization time, a sampling was carried out ranging from 1 to 24 h by means of confocal microscopy control (Supplementary Materials Figure S20) that showed that at all times, there was a gradual entry of cells as a function of time, which was more evident in the fractions that went from 8 to 24 h.

With regard to the treatment of BJ-hTERT cells with Au@16-Ph-16/DNA–5-Fu-compacted nanosystems (Figure 13), nanoparticles were observed on the cells at 0 h in a very small proportion (Figure 13A–D), being more evident 24 h after the treatment was carried out (Figure 13E–H). Structures compatible with nanosystems were also observed outside the cell, again more evident at 24 h (Figure 13F,H). Note that as was the case with the free nanoparticle treatment, it is difficult to say with certainty whether the nanoparticles were still entering the cell or some quantity of them was being expelled out of them. We could also observe that the morphology of some cells at 24 h was rounded (Figure 13F–H), which may constitute evidence of the involvement of these cells by treatment with the nanosystem; in some areas, what could be remnants of cytoplasm or degraded cells could be seen. Thus, in conclusion, the TEM and confocal microscopies showed that Ci and Ni nanoparticles were effectively internalized and delivered to different cellular compartments. However, the differential uptake among different formulations may have been related to differences in size, surface charge, and aggregation state, which influenced colloidal stability in biological media.

## 4. Conclusions

Chemotherapeutic agents like 5-Fu are used as treatment against malignant neoplasia’s principal multiple secondary effects and derived sequelae. In this study, different formulations of gold nanoparticles containing the 5-Fu anticancer drug Au@16-Ph-16/DNA–5-Fu (C_i_) were successfully produced from Au@16-Ph-16 precursor nanoparticles (Ni), which could transport different concentrations of 5-Fu to the cell. To this end, prior conformational studies by using AFM, CD, and fluorescence spectroscopy guaranteed the full compaction of the DNA–5-Fu complex induced by the Au@16-Ph-16 precursor at an optimal relative molar ratio of R = C_Au@16-Ph-16_/C_DNA._ Importantly, the synthesized nanosystems were highly charged, well-dispersed, and stabilized in water at a low ionic strength, as demonstrated by the zeta potential, UV–visible spectroscopy, and DLS results. Moreover, the Ci nanoparticles were produced with a high loading efficiency and could more rapidly be released by using the appropriate Ni precursor concentration. These findings are crucial to guarantee their possible use for in vivo delivery of the 5-Fu drug and to ensure the efficacy of cancer treatment. More specifically, in connection with the physicochemical characterization of the explored N_i_ and C_i_ nanosystems explored, TEM images and confocal studies revealed that the internalization of highly charged and smaller-sized N_3_ and C_3_ formulations induced a better cell degradation in lung cancer cells compared to the other samples. Moreover, a moderate selectivity of the synthesized nanosystems against cancer cells vs. healthy cells was observed using the MTT assay. The outcomes of the present study are also significant because they delineate new avenues for a fresh strategy to induce drug release from a compacted nanosystem after internalization in cancer cells. Namely, when the adequate dose of Au@16-Ph-16 precursor was added to the effectively internalized transporter, biopolymer decompaction was induced by 5-Fu drug release in the target site, contributing to increased effectiveness of the treatment and thereby reducing the possible side effects of drug administration. These findings suggest that synthetized nanoparticles are effective and promising anticancer drug carriers to be applied in lung cancer therapy.

## Figures and Tables

**Table 1:** Drug loading and release parameters for different formulations of Au@16-Ph-16/DNA–5-Fu-compacted nanosystems.

Nanosystem	Drug Loading (%)	Drug Release (%)	*t*_1/2_ Release (Min)
C_1_	97%	94%	1.0 min
C_2_	95%	98 %	3.98 min
C_3_	96%	99 %	6.3 min

**Table 2:** Zeta potential and dynamic light scattering (DLS) size distribution by number of Au@16-Ph-16 precursors and Au@16-Ph-16/DNA–5-Fu-compacted nanosystems at different concentrations in water and phosphate-buffered saline (PBS) 0.1× (ionic strength = 1.63 mM and pH = 7.4). Abbreviations: 19× and 1× correspond to 19× and 1× preparations for Ci and Ni formulations, respectively.

System	Zeta Potential/mV in Water (19×)	Zeta Potential/mV in PBS 0.1× (19×)	Diameter/nm in Water (19×)	Diameter/nm in PBS 0.1× (19×)	Diameter/nm in PBS 0.1× (1×)
N_1_	(30 ± 3)	(18 ± 2)	(13 ± 4)	(23 ± 2)	(23 ± 2)
N_2_	(35 ± 3)	(28 ± 3)	(10 ± 2)	(21 ± 3)	(23 ± 3)
N_3_	(40.0 ± 1.0)	(37.0 ± 1.0)	(9 ± 2)	(10 ± 3)	(13 ± 3)
C_1_	(−30 ± 3)	(−45 ± 8)	(36 ± 6)	(34 ± 7)	d_1_ = (38 ± 16); 29.7% d_2_ = (106 ± 13); 70.3%
C_2_	(−29 ± 6)	(−46 ± 6)	(88 ± 6)	(85 ± 5)	d_1_ = (41 ± 19); 99.6% d_2_ = (255 ± 30); 0.4%
C_3_	(−33 ± 3)	(−56 ± 5)	(16 ± 4)	(14 ± 3)	(26 ± 5)

## Data Availability

Data is contained within the article or Supplementary Materials.

## Supplementary Materials

The following are available online at https://www.mdpi.com/1999-4923/13/3/423/s1. Table S1. Absorbance of the nanocomplexes over the time at the SPR maximum. Figure S1: Characterization and chemical structure of 16-Ph-16 gemini surfactant. Figure S2: Stability of N_i_ and C_i_ formulations. Figure S3: Loading assay for C_i_ nanocomplexes. Figure S4: Release assay for C_i_ nanocomplexes. Figure S5: Release profile of 5-Fu from Ci-compacted nanosystems. Figure S6: TEM image and size distribution of Au@16-Ph-16 nanoparticles. Figure S7: EDS spectrum of Au@16-Ph-16 nanoparticles. Figure S8: Fluorescence spectra of DNA/5-Fu complex in the presence of Au@16-Ph-16 nanoparticles. Figure S9: AFM topography image of DNA in extended-coil conformation. Figures S10 and S11: Zeta potential of Au@16-Ph-16 and Au@16-Ph-16/DNA–5-Fu nanosystems in water at the 19× condition. Figures S12 and S13: DLS size distribution by number of Au@16-Ph-16 and Au@16-Ph-16/DNA–5-Fu nanosystems in water at the 19× condition. Figures S14 and S15: Zeta potential of Au@16-Ph-16 and Au@16-Ph-16/DNA–5-Fu nanosystems in PBS 0.1× (I = 1.63 mM and pH = 7.4) at the 19× condition. Figures S16, S17, S18 and S19: DLS size distribution by number of Au@16-Ph-16 and Au@16-Ph-16/DNA–5-Fu nanosystems in PBS 0.1× (I = 1.63 mM and pH = 7.4) at the 19× and 1× conditions. Figure S20. Confocal photomicrographs showing control of internalization of nanosystems in cells over time.
