# Supplementary material for: Biocompatible DNA/5-Fluorouracil-Gemini Surfactant-Functionalized Gold Nanoparticles as Promising Vectors in Lung Cancer Therapy
Source: Pharmaceutics. 2021 Mar 21;13(3):423. doi: 10.3390/pharmaceutics13030423 (PMC8004209; doi:10.3390/pharmaceutics13030423)
Supplement: Supplementary file 1 [file pharmaceutics-13-00423-s001.pdf]

pharmaceutics

Supplementary Material: Biocompatible DNA/5-Fluorouracil-Gemini Surfactant-Functionalized Gold Nanoparticles as Promising Vectors in Lung Cancer Therapy

Rosa M. Giráldez-Pérez, Elia Grueso, Inmaculada Domínguez, Nuria Pastor, Edyta Kuliszewska, Rafael Prado-Gotor and Francisco Requena-Domenech

Supplementary Material S1. Characterization of p-16-Ph-16 cationic gemini surfactant

NMR Characterization

N,N′-Di-n-hexadecyl-*N,N,N′,N′*-tetramethyl-phenylene-1,4-dimethylenammonium dibromide (p-16-Ph-16). ^{1}H-NMR (400 MHz, $CDCl_3$, δ in ppm): 7.79 (s,br, 4 H, H-2 (4x)); 5.10 (s,br, 4 H, H-1′ (2x)); 3.51 (s,br, 4 H, H-1″ (2x)); 3.21 (s,br, 12 H, CH_3 (4x)); 1.80 (s,br, 4 H, H-2″ (2x)); 1.35 (s,br, 4 H, H-3″ (2x)); 1.24$_c$ (m,br, 48 H, H-4″ to H-15″ (2x)); 0.68 (t, 6 H, J=7.0 Hz, H-16″ (2x)). ^{13}C NMR (100 MHz, $CDCl_3$, δ in ppm): 133.6 (d, C-2 (4x)); 128.7 (s, C-1 (2x)); 66.6 (t, C-1′ (2x)); 65.1 (t, C-1″ (2x)); 49.6 (q, CH_3 (4x)); 31.4 (t, C-14″ (2x)); 29.7-29.4 (t (10x), C-4″ to C-13″ (2x)); 28.5 (t, C-3″ (2x)); 26.4 (t, C-2″ (2x)); 22.7 (t, C-15″ (2x)); 14.1 (q, C-16″ (2x)).

Citation: Giráldez-Pérez, R.M.; Grueso, E.; Domínguez, I.; Pastor, N.; Kuliszewska, E.; Prado-Gotor, R.; Requena-Domenech, F. Biocompatible DNA/5-Fluorouracil-Gemini Surfactant-Functionalized Gold Nanoparticles as Promising Vectors in Lung Cancer Therapy. *Pharmaceutics* **2021**, *13*, 423. https://doi.org/10.3390/pharmaceutics13030423

Publisher's Note: MDPI stays neutral with regard to jurisdictional claims in published maps and institutional affiliations.

Copyright: © 2021 by the authors. Licensee MDPI, Basel, Switzerland. This article is an open access article distributed under the terms and conditions of the Creative Commons Attribution (CC BY) license (http://creativecommons.org/licenses/by/4.0/).

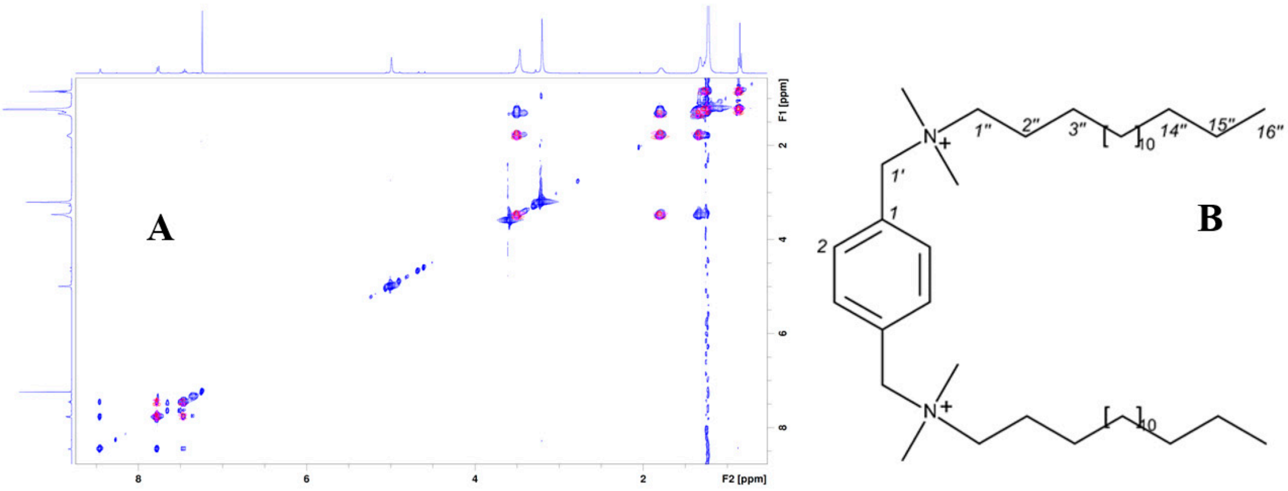

Figure S1. (**A**) Combined COSY (pinc) and TOCSY (blue) spectra of compound m-16-Ph-16 together with the respective ^{1}H NMR spectrum on both axes. (**B**) Structure of the compound p-16-Ph-16. The numbering is accordance to those used in for the NMR shift data. It is not in accordance to the IUPAC nomenclature, but allows an easy comparison of different shifts in the compound.

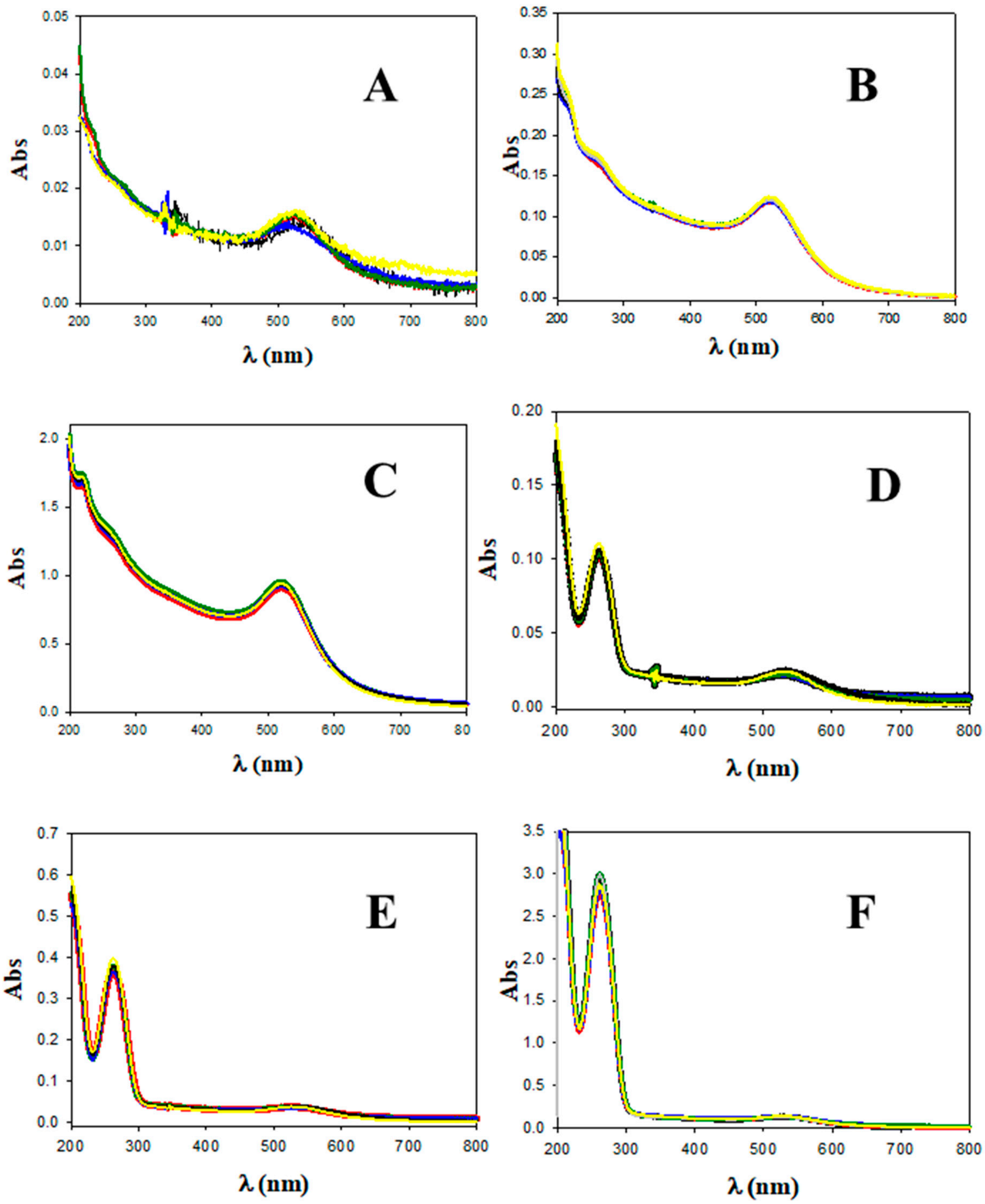

Figure S2. Stability for different formulations over time: 1 h (in grey); 48 h (in green); 72 h (in red); 5 days (in blue); 1 week (in black); 2 weeks (in yellow). (**A**) N_1; (**B**) N_2; (**C**) N_3; (**D**) C_1; (**E**) C_2 and (**F**) C_3.

Table S1. Stability of the nanosystems over time after preparation of different formulations. Absorbance measurements were carried out at the maximum SPR band of gold nanosystem in each case.

Nanosystem	1 h	48 h	72 h	5 days	1 week	2 weeks
	$\lambda_{max,SPR}$/ $Abs_{max,SPR}$	$\lambda_{max,SPR}$/ $Abs_{max,SPR}$	$\lambda_{max,SPR}$/ $Abs_{max,SPR}$	$\lambda_{max,SPR}$/ $Abs_{max,SPR}$	$\lambda_{max,SPR}$/ $Abs_{max,SPR}$	$\lambda_{max,SPR}$/ $Abs_{max,SPR}$
N_1	519 nm/ 0.0155	519 nm/ 0.0153	519 nm/ 0.0149	518 nm/ 0.0137	518 nm/ 0.0137	519 nm/ 0.0142
N_2	519 nm/ 0.1201	519 nm/ 0.1222	519 nm/ 0.1176	519 nm/ 0.1173	519 nm/0.1176	519 nm/0.1235
N_3	520 nm/ 0.9387	520 nm/ 0.9611	520 nm/ 0.9140	520 nm/ 0.9293	520 nm/ 0.9314	520 nm/0.9407
C_1	527 nm/ 0.0226	527 nm/ 0.0218	527 nm/ 0.0207	527 nm/ 0.0216	527 nm/ 0.0217	527 nm/ 0.0239
C_2	529 nm/ 0.0377	529 nm/ 0.0380	529 nm/ 0.0372	529 nm/ 0.0345	529 nm/ 0.0394	529 nm/ 0.0379
C_3	529 nm/0.1271	530 nm/ 0.1277	530 nm/ 0.1292	529 nm/ 0.1369	530 nm/ 0.1327	530 nm/ 0.1364

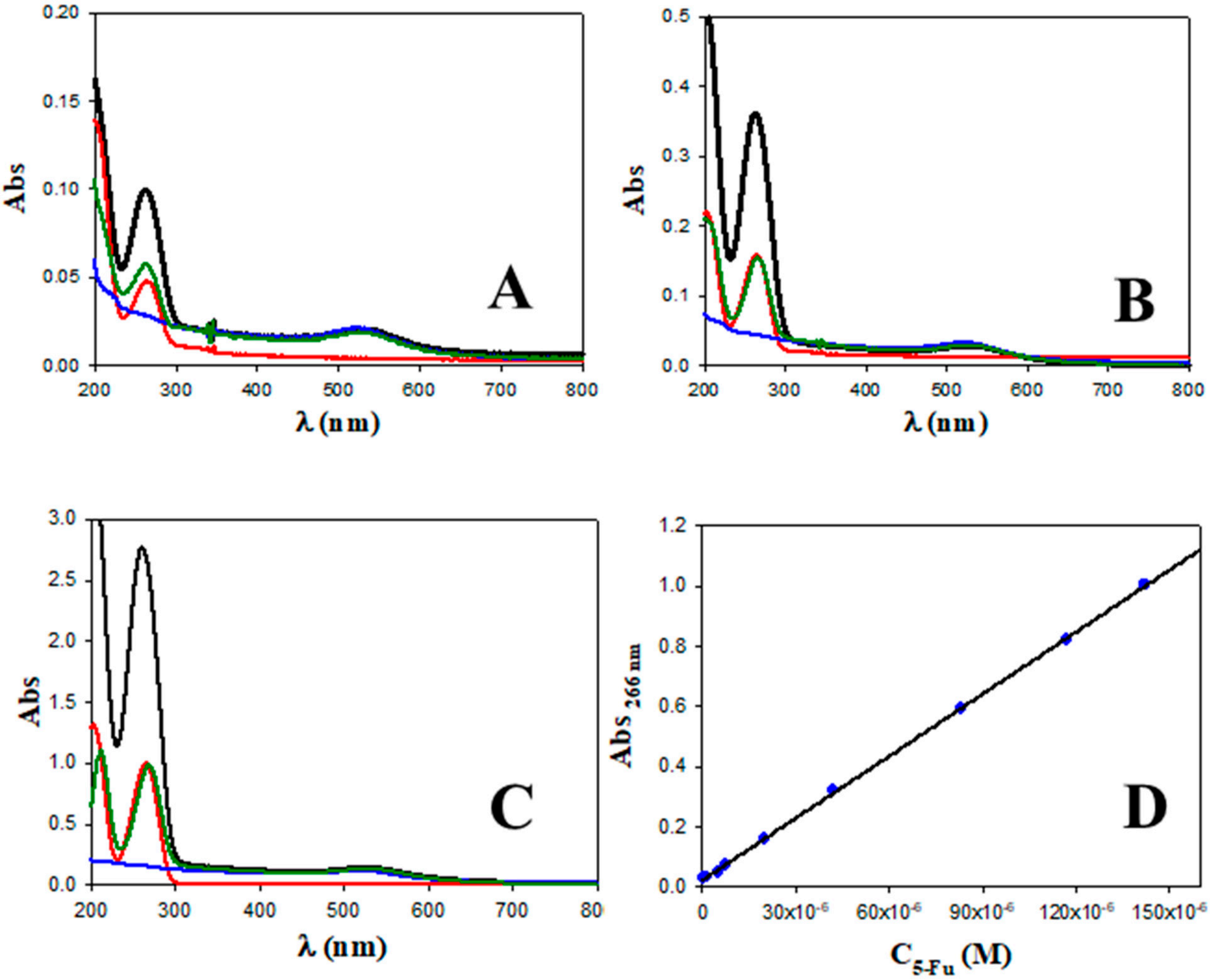

Figure S3. Loading assay for C_i nanocomplexes. The UV-vis spectra recorded on the solution of 5-Fu was measured before and after complexation in nanoparticles and 1 h of stabilization and dialysis. All the formulations were prepared at 19X concentrations to easily visualization of the spectra (see section 2.1.2 and 2.1.3 of the paper for more details). (**A**) free 5-Fu,$_1$ drug in C_1 formulation (in red); DNA corrected C_1 spectrum for C_1 formulation (in green); N_1 spectrum for C_1 formulation (in blue); C_1 formulation. (**B**) free 5-Fu,$_2$ drug in C_2 formulation (in red); DNA corrected C_2 spectrum for C_2 formulation (in green); N_2 spectrum for C_2 formulation (in blue); C_2 formulation. (**C**) free 5-Fu,$_3$ drug in C_3 formulation (in red); DNA corrected C_3 spectrum for C_3 formulation (in green); N_3 spectrum for C_3 formulation (in blue); C_3 formulation. (**D**) 5-Fu calibration curve used for measuring loaded-drug in different C_i formulations.

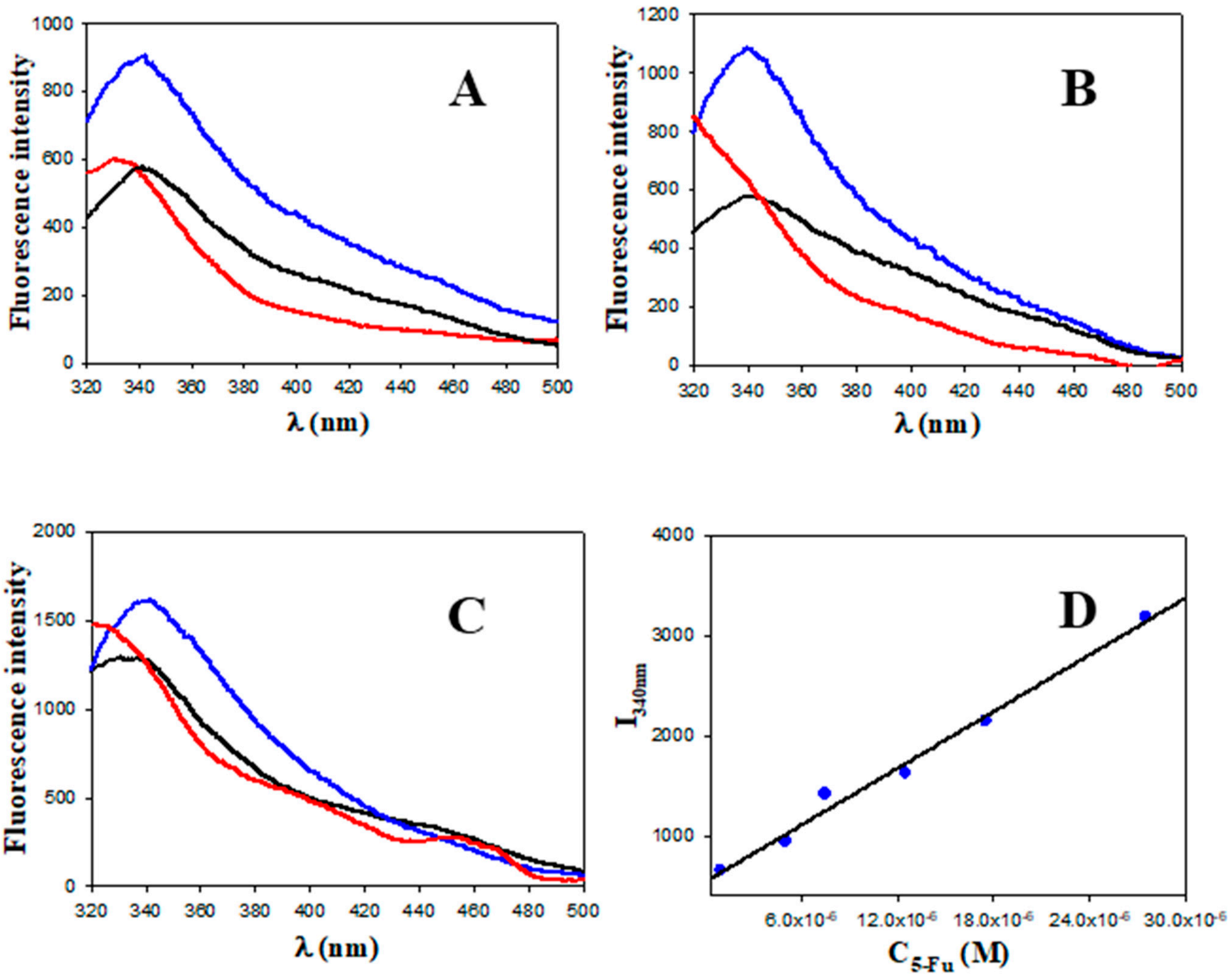

Figure S4. Release assay for C_i nanocomplexes. The spectra of C_1 + N_1 systems are displayed immediately after mixing (in blue) and after stabilization of the nanosystem and complete 5-Fu release (in red). The fluorescence spectra recorded on the solution of free 5-Fu (in black) was measured for comparative purpose. All the formulations were prepared as described in section 2.1.2 and 2.1.3 of the paper. (**A**) C_1 release in the presence of N_1 nanoparticles; (**B**) C_2 release in the presence of N_2 nanoparticles; (**C**) C_3 release in the presence of N_3 nanoparticles; (**D**) 5-Fu calibration curve used for measuring released-drug in different C_i formulations.

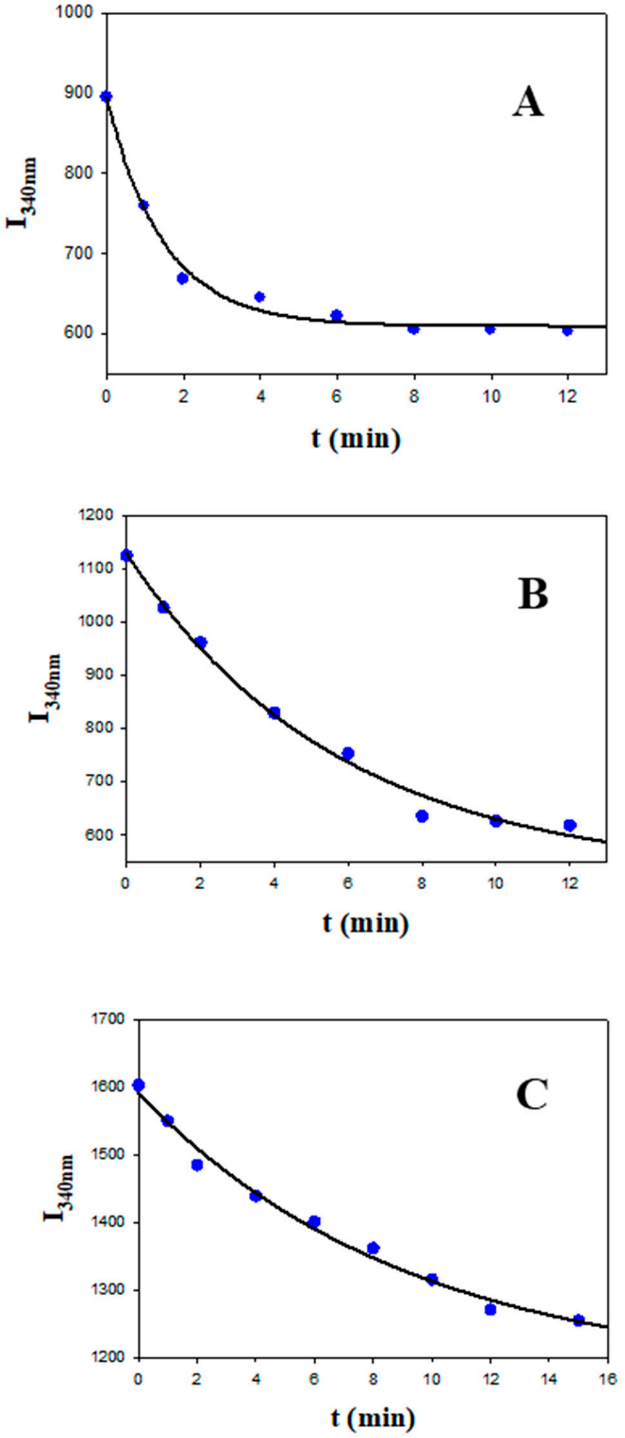

Figure S5. In vitro release profile of 5-Fu from Ci compacted nanosystems upon addition of Ni formulations. (**A**) 5-Fu release from C_1; (**B**) 5-Fu release from C_2; (**C**) 5-Fu release from C_3.

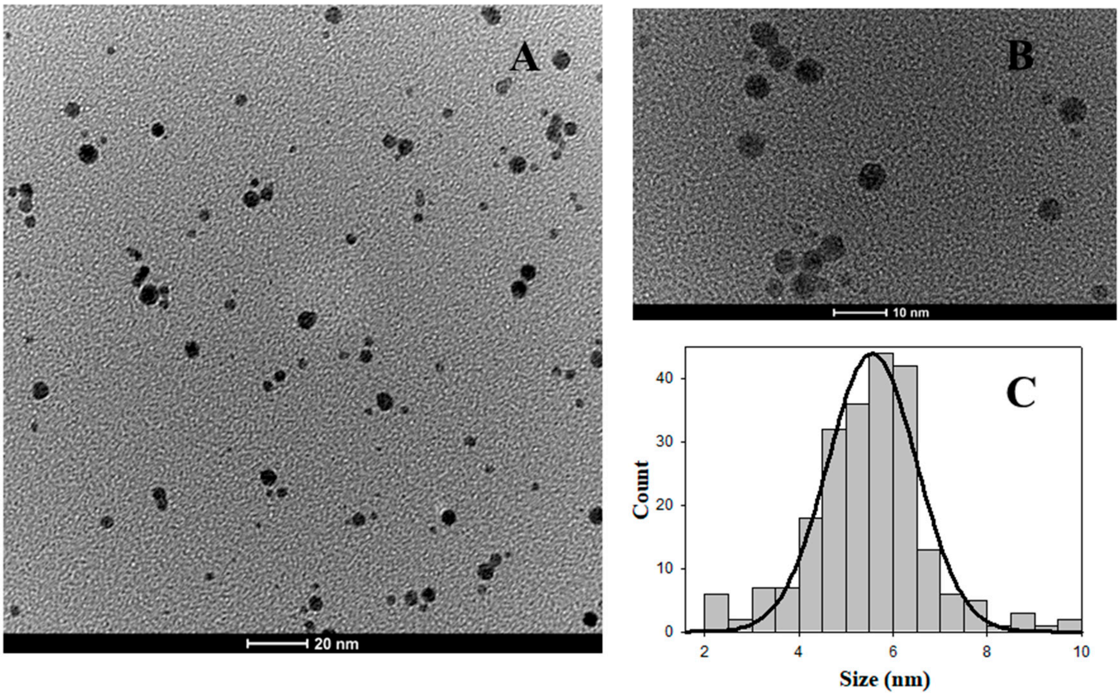

Figure S6. (**A**,**B**) TEM image of free Au@16-Ph-16 gold nanoparticles. (**C**) Size distribution of Au@16-Ph-16 in water.

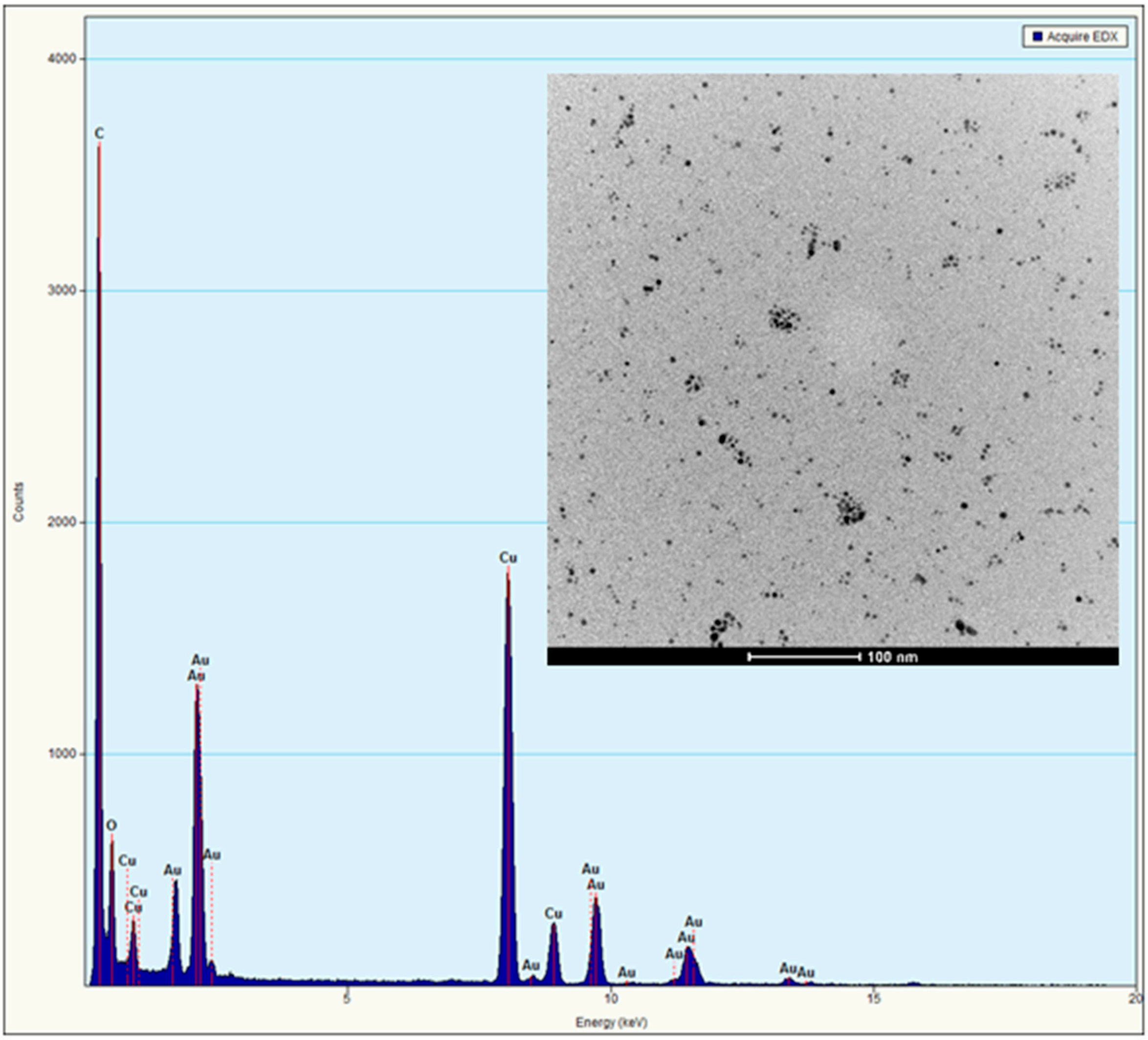

Figure S7. EDS spectrum of Au@16-Ph-16 nanoparticles. Elemental mapping results indicate the distribution of Gold element on the particles. Bear in mind that the presence of copper ion is due to the use of copper grid coated with a carbon film needed for TEM measurement.

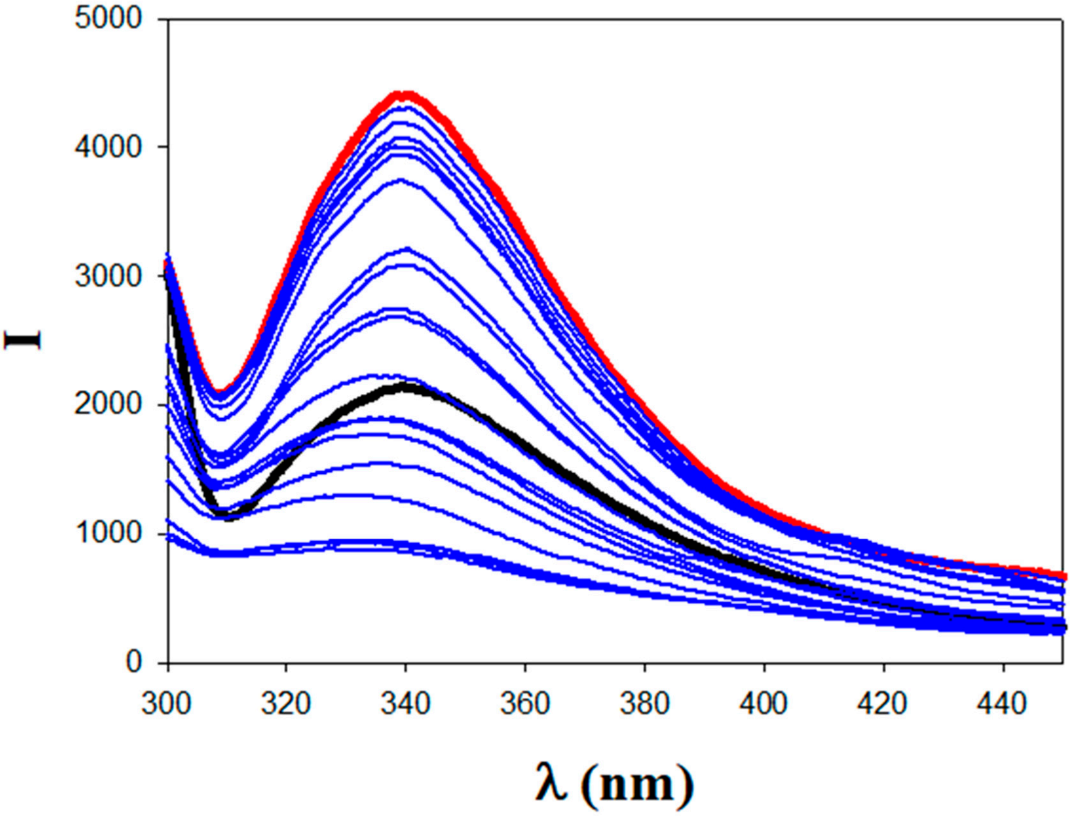

Figure S8. The emission spectra of 5-Fu alone and DNA/5-Fu in the absence and the presence of Au@16-Ph-16. Black spectrum corresponds to 5-Fu alone. Red spectrum corresponds to DNA/5-Fu complex in the absence of gold nanoparticles. Blue spetra corresponds to DNA/5-Fu titration at different gold nanoparticle concentrations. $C_{5\text{-Fu}}$ = 41.7 µM and C_{DNA} = 50.0 µM were fixed, and the precursor concentration $C_{Au@16\text{-}Ph\text{-}16}$ was ranged from 0.187 to 51.6 nM in blue spectra.

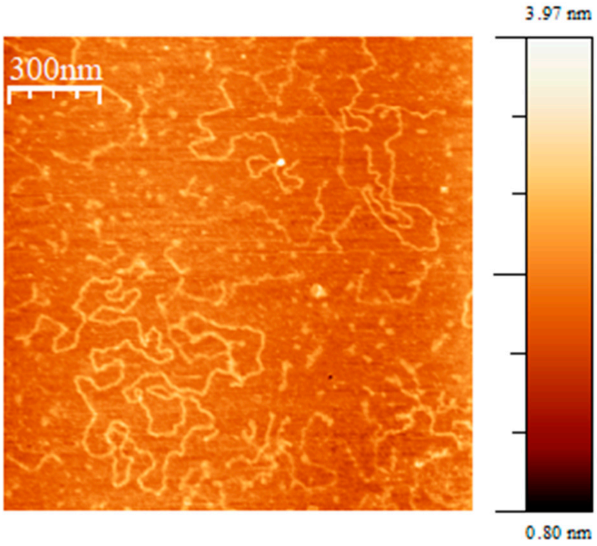

Figure S9. AFM topography image of DNA in extended-coil conformation adsorbed on APTES modified mica surface in cacodylate buffer (ionic strength = 1.63 mM, pH = 7.4), C_{DNA} = 0.3 µM.

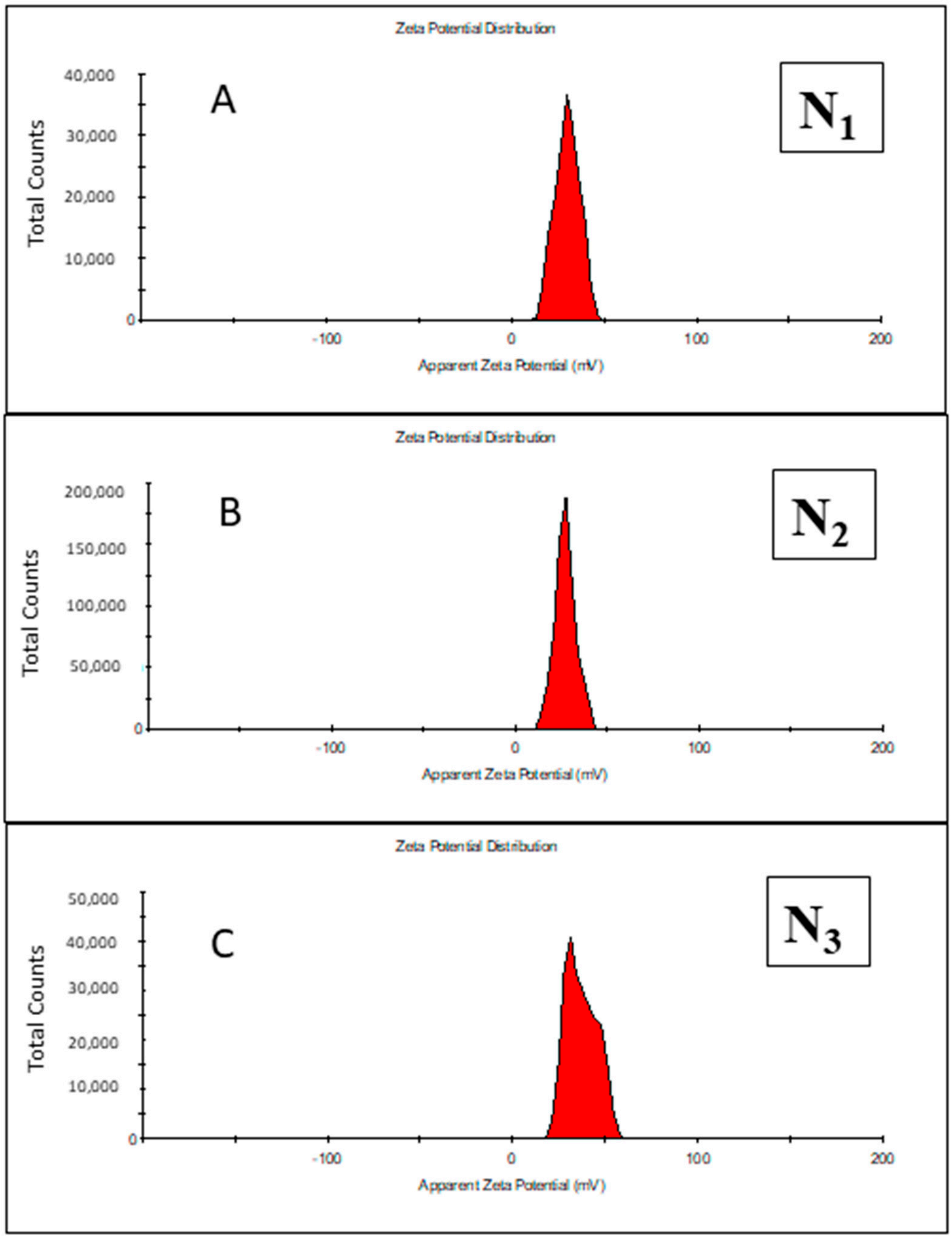

Figure S10. Zeta potential of Au@16-Ph-16 nanoparticles at different $C_{Au@16\text{-}Ph\text{-}16}$ concentrations in water. The three Au@16-Ph-16 formulations designated as N_i in abbreviated form. (**A**) N_1, $C_{Au@16\text{-}Ph\text{-}16}$ = 1.94 nM; (**B**) N_2, $C_{Au@16\text{-}Ph\text{-}16}$ = 7.69 nM; (**C**) N_3, $C_{Au@16\text{-}Ph\text{-}16}$ = 54 nM. All the samples were prepared at 19X concentration (19 times concentrated respect to that described).

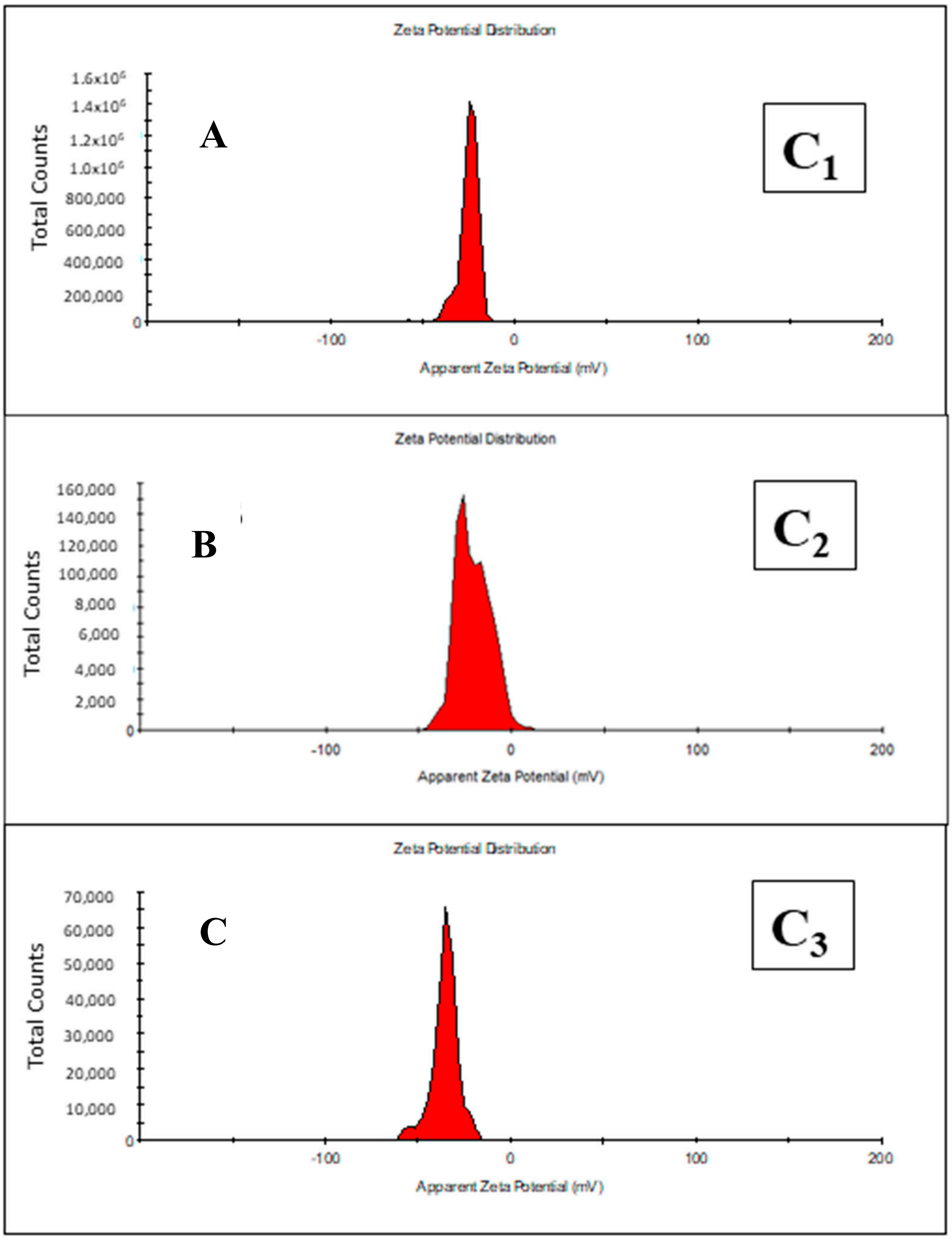

Figure S11. Zeta potential of Au@16-Ph-16/DNA-5-Fu compacted nanosystems for different formulations in water. The three Au@16-Ph-16/DNA-5-Fu formulations designated as C_i in abbreviated form. (**A**) C_1, $C_{5\text{-Fu}}$ = 0.25 μM, C_{DNA} = 0.30 μM, and $C_{Au@16\text{-Ph-}16}$ = 0.011 nM; (**B**) C_2, $C_{5\text{-Fu}}$ = 1.00 μM, C_{DNA} =1.19 μM, and $C_{Au@16\text{-Ph-}16}$ = 0.045 nM; and (**C**) C_3, $C_{5\text{-Fu}}$ = 7.5 μM, C_{DNA} = 8.9 μM, and $C_{Au@16\text{-Ph-}16}$ = 0.33 nM. All the samples were prepared at 19X concentration (19 times concentrated respect to that described).

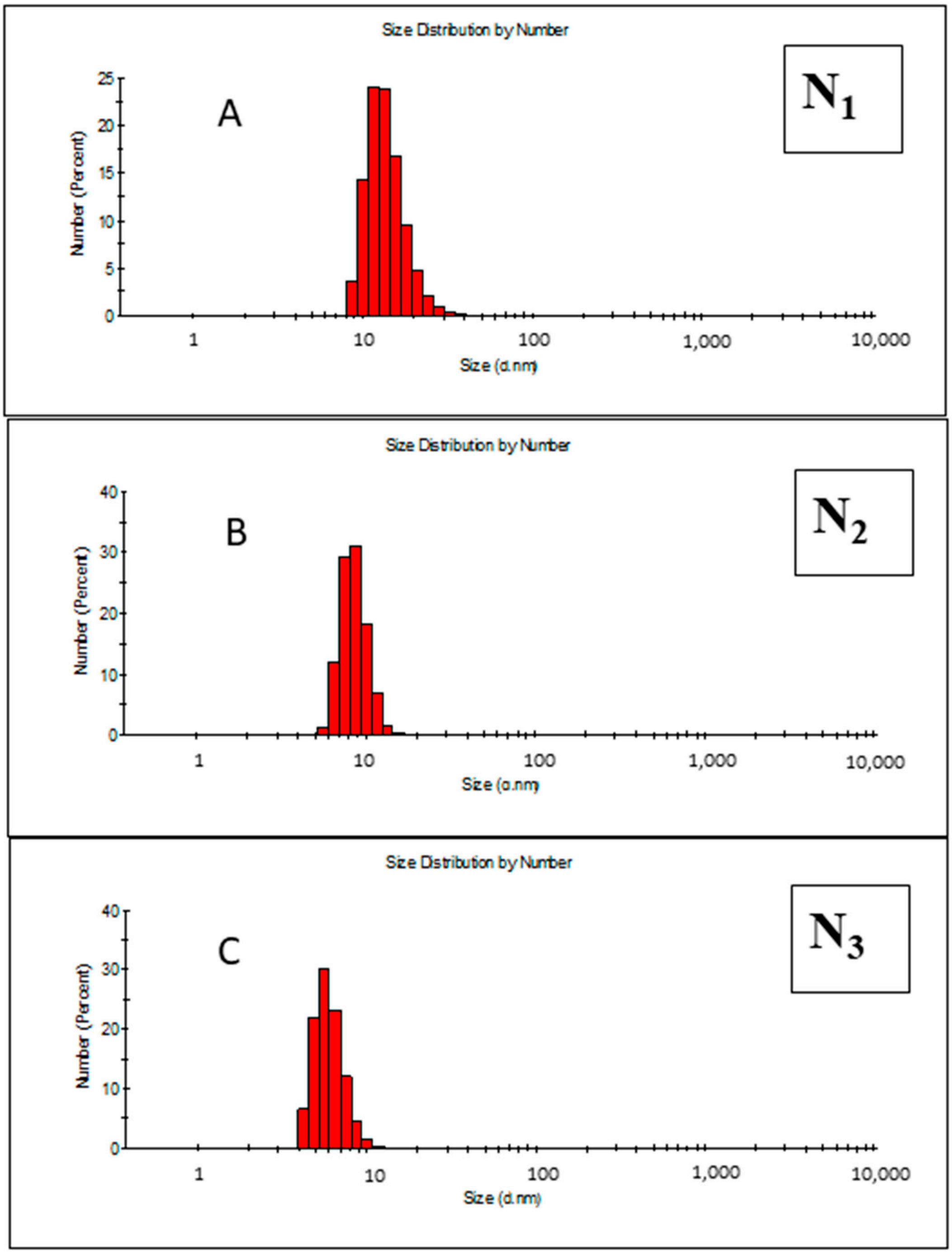

Figure S12. DLS size distribution by number of Au@16-Ph-16 nanosystems at different $C_{Au@16\text{-}Ph\text{-}16}$ concentrations in water. The three Au@16-Ph-16 formulations designated as N_i in abbreviated form. (**A**) N_1, $C_{Au@16\text{-}Ph\text{-}16}$ = 1.94 nM; (**B**) N_2, $C_{Au@16\text{-}Ph\text{-}16}$ = 7.69 nM; (**C**) N_3, $C_{Au@16\text{-}Ph\text{-}16}$ = 54 nM. All the samples were prepared at 19X concentration (19 times concentrated respect to that described).

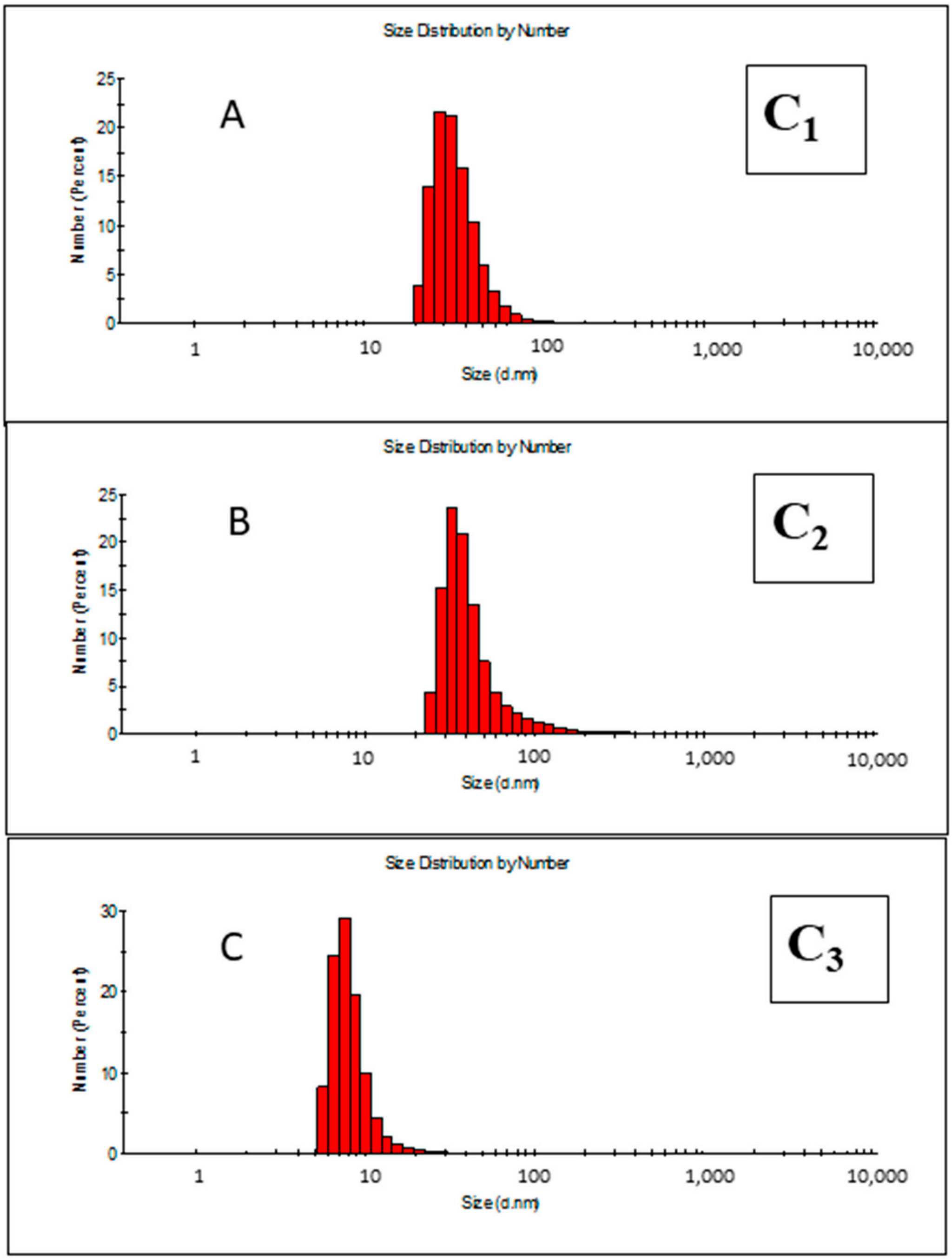

Figure S13. DLS size distribution by number of Au@16-Ph-16/DNA-5-Fu compacted nanosystems for different formulations in water. The three Au@16-Ph-16/DNA-5-Fu formulations designated as C_i in abbreviated form. (**A**) C_1, $C_{5\text{-Fu}}$ = 0.25 μM, C_{DNA} = 0.30 μM, and $C_{Au@16\text{-Ph-}16}$ = 0.011 nM; (**B**) C_2, $C_{5\text{-Fu}}$ = 1.00 μM, C_{DNA} =1.19 μM, and $C_{Au@16\text{-Ph-}16}$ = 0.045 nM; and (**C**) C_3, $C_{5\text{-Fu}}$ = 7.5 μM, C_{DNA} = 8.9 μM, and $C_{Au@16\text{-Ph-}16}$ = 0.33 nM. All the samples were prepared at 19X concentration (19 times concentrated respect to that described).

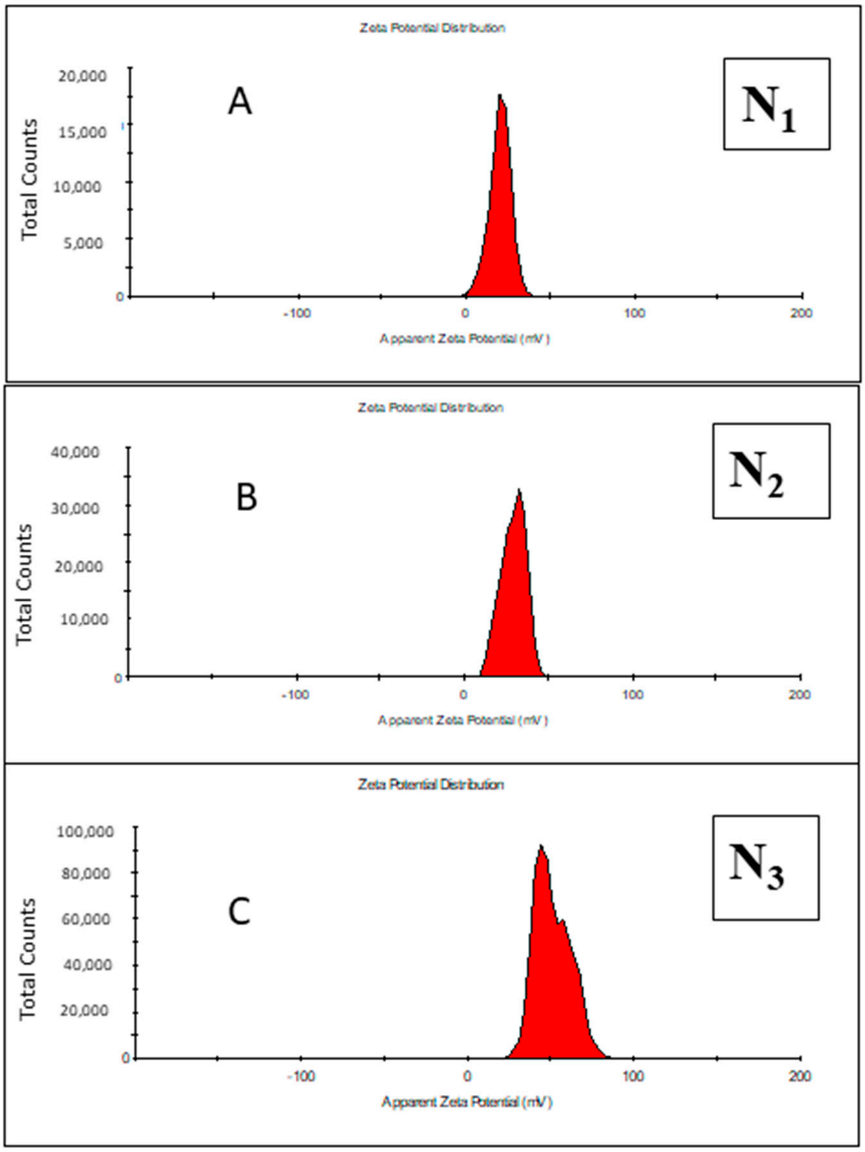

Figure S14. Zeta potential of Au@16-Ph-16 nanoparticles at different $C_{Au@16\text{-}Ph\text{-}16}$ concentrations in PBS solution diluted at 0.1x concentration (ionic strength = 1.63 mM, pH = 7.4). The three Au@16-Ph-16 formulations designated as N_i in abbreviated form. (**A**) N_1, $C_{Au@16\text{-}Ph\text{-}16}$ = 1.94 nM; (**B**) N_2, $C_{Au@16\text{-}Ph\text{-}16}$ = 7.69 nM; (**C**) N_3, $C_{Au@16\text{-}Ph\text{-}16}$ = 54 nM. All the samples were prepared at 19X concentration (19 times concentrated respect to that described).

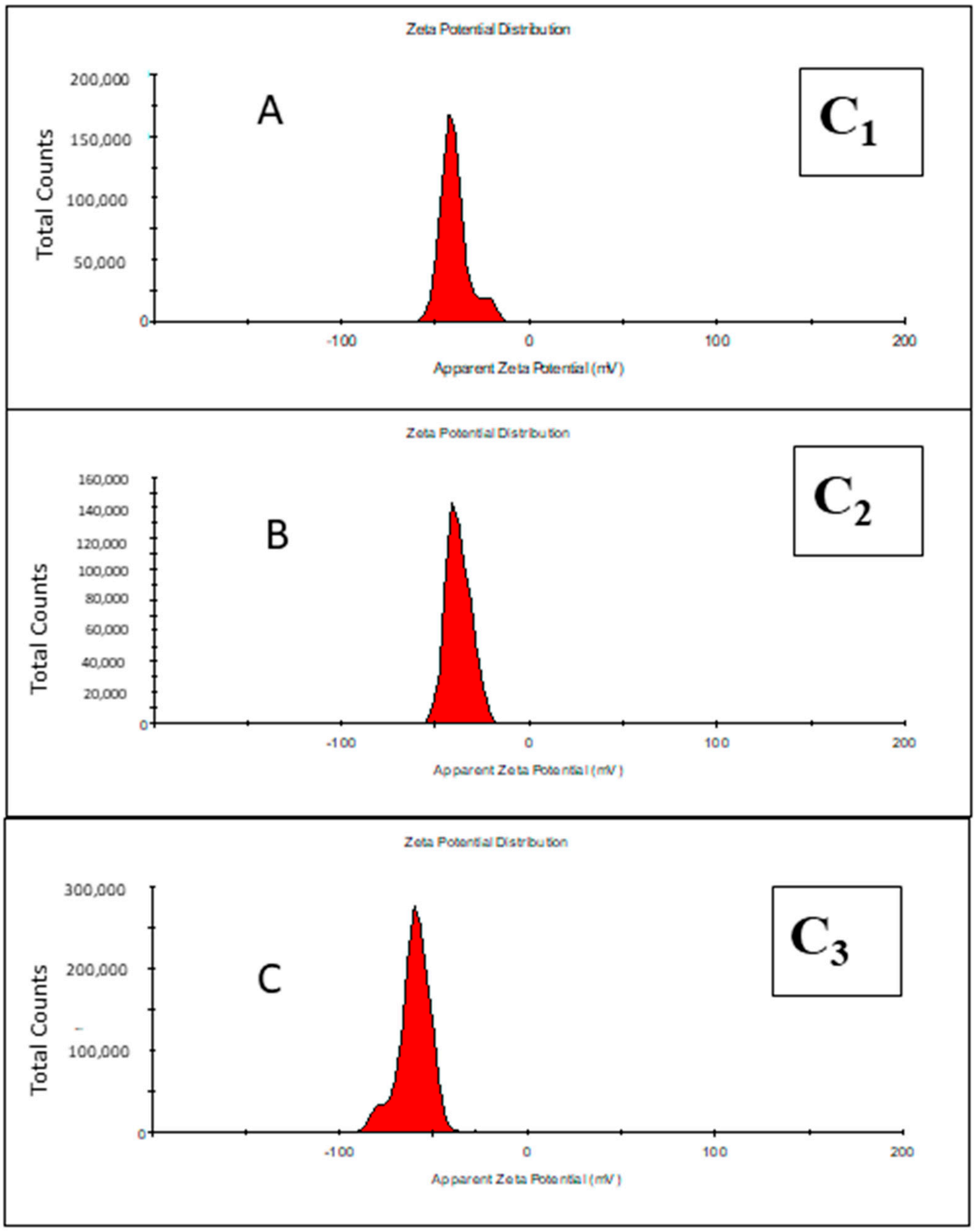

Figure S15. Zeta potential of Au@16-Ph-16/DNA-5-Fu compacted nanosystems for different formulations in PBS solution diluted at 0.1x concentration (ionic strength = 1.63 mM, pH = 7.4). The three Au@16-Ph-16/DNA-5-Fu formulations designated as C_i in abbreviated form. (**A**) C_1, $C_{5\text{-Fu}}$ = 0.25 µM, C_{DNA} = 0.30 µM, and $C_{Au@16\text{-Ph-}16}$ = 0.011 nM; (**B**) C_2, $C_{5\text{-Fu}}$ = 1.00 µM, C_{DNA} =1.19 µM, and $C_{Au@16\text{-Ph-}16}$ = 0.045 nM; and (**C**) C_3, $C_{5\text{-Fu}}$ = 7.5 µM, C_{DNA} = 8.9 µM, and $C_{Au@16\text{-Ph-}16}$ = 0.33 nM. All the samples were prepared at 19X concentration (19 times concentrated respect to that described).

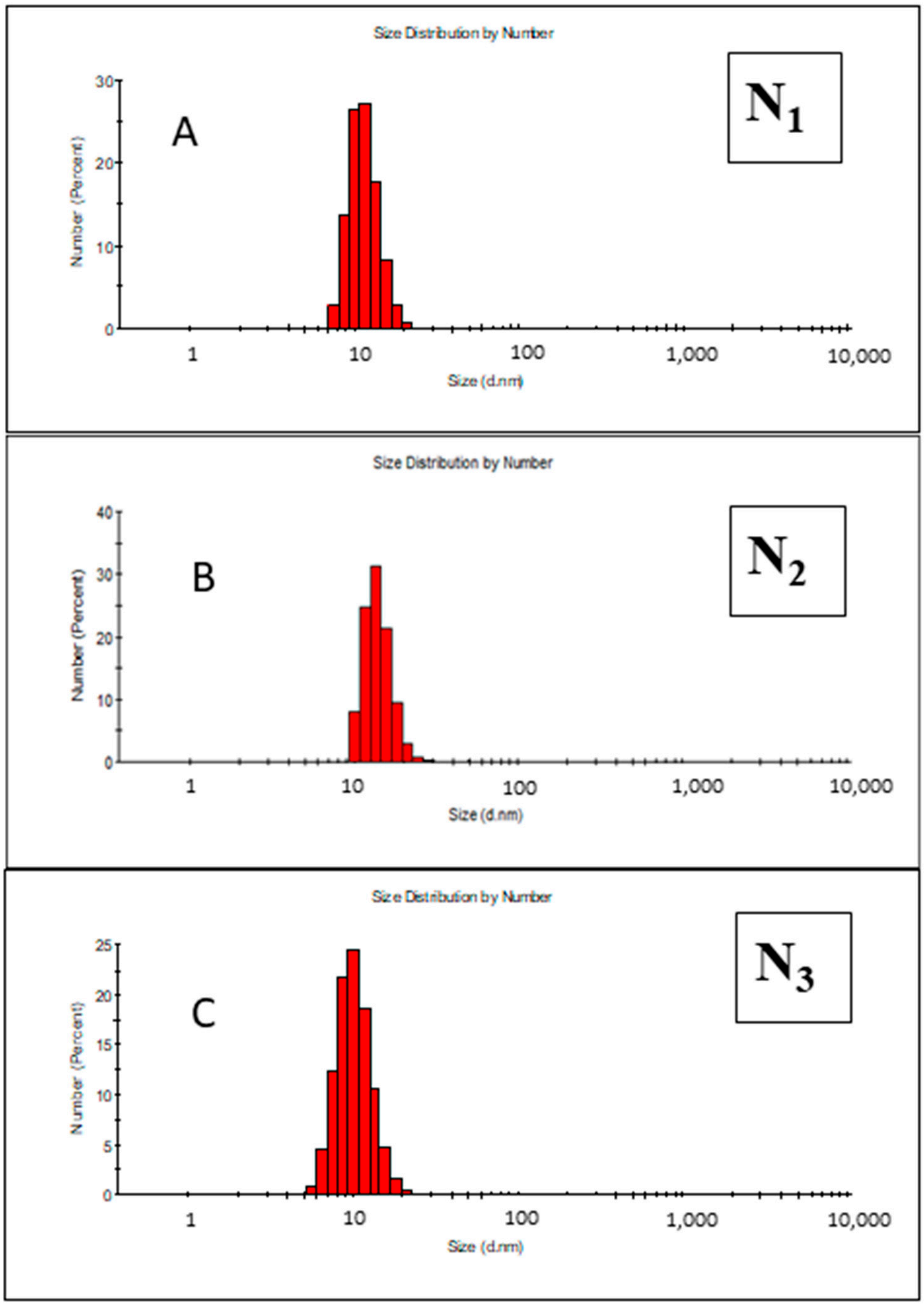

Figure S16. DLS size distribution by number of Au@16-Ph-16 nanosystems at different $C_{Au@16\text{-}Ph\text{-}16}$ concentrations in PBS solution diluted at 0.1x concentration (ionic strength = 1.63 mM, pH = 7.4). The three Au@16-Ph-16 formulations designated as N_i in abbreviated form. (**A**) N_1, $C_{Au@16\text{-}Ph\text{-}16}$ = 1.94 nM; (**B**) N_2, $C_{Au@16\text{-}Ph\text{-}16}$ = 7.69 nM; (**C**) N_3, $C_{Au@16\text{-}Ph\text{-}16}$ = 54 nM. All the samples were prepared at 19X concentration (19 times concentrated respect to that described).

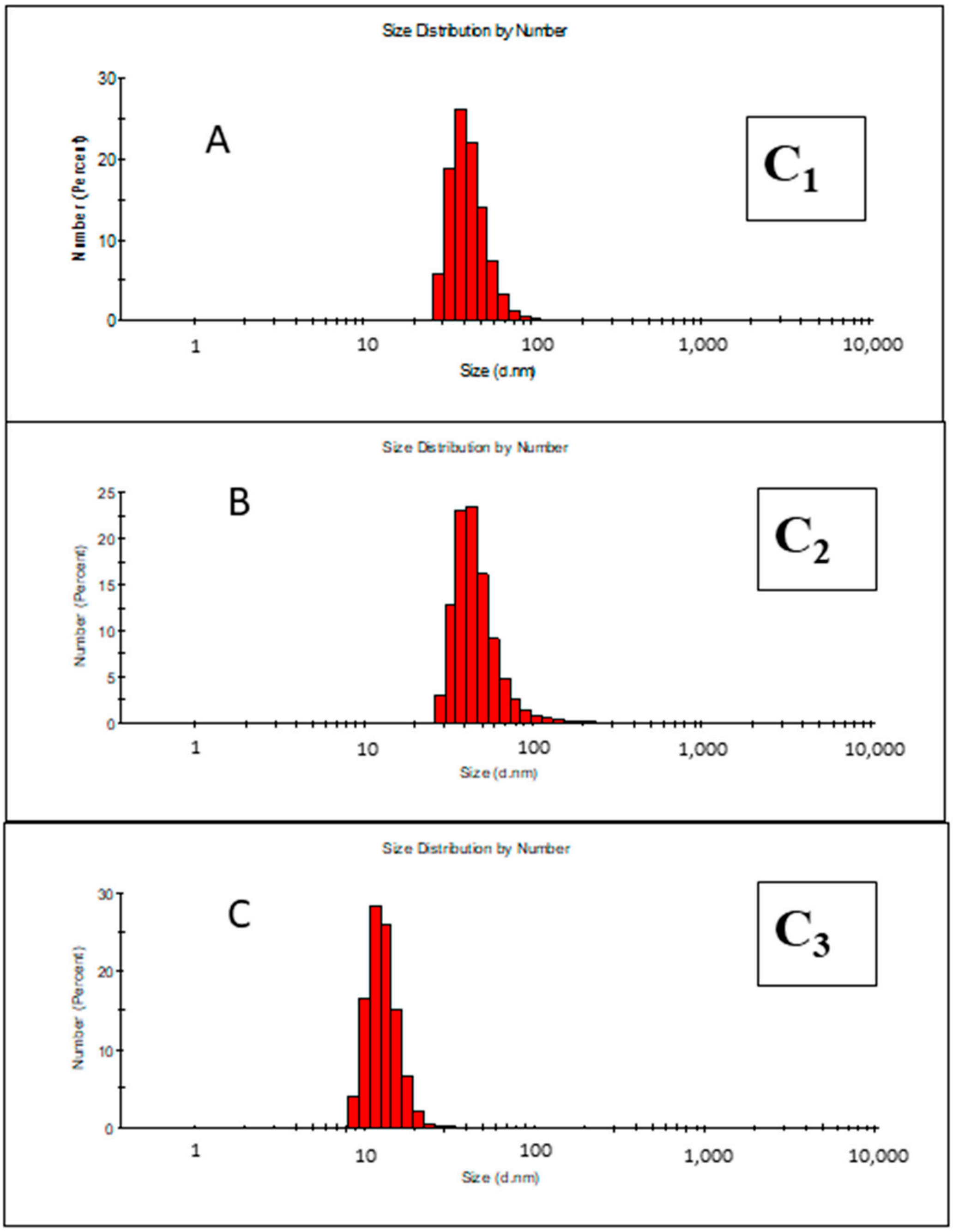

Figure S17. DLS size distribution by number of Au@16-Ph-16/DNA-5-Fu compacted nanosystems in PBS solution diluted at 0.1x concentration (ionic strength = 1.63 mM, pH = 7.4). The three Au@16-Ph-16/DNA-5-Fu formulations designated as C_i in abbreviated form. (**A**) C_1, $C_{5\text{-Fu}}$ = 0.25 µM, C_{DNA} = 0.30 µM, and $C_{Au@16\text{-Ph-}16}$ = 0.011 nM; (**B**) C_2, $C_{5\text{-Fu}}$ = 1.00 µM, C_{DNA} =1.19 µM, and $C_{Au@16\text{-Ph-}16}$ = 0.045 nM; and (**C**) C_3, $C_{5\text{-Fu}}$ = 7.5 µM, C_{DNA} = 8.9 µM, and $C_{Au@16\text{-Ph-}16}$ = 0.33 nM. All the samples were prepared at 19X concentration (19 times concentrated respect to that described).

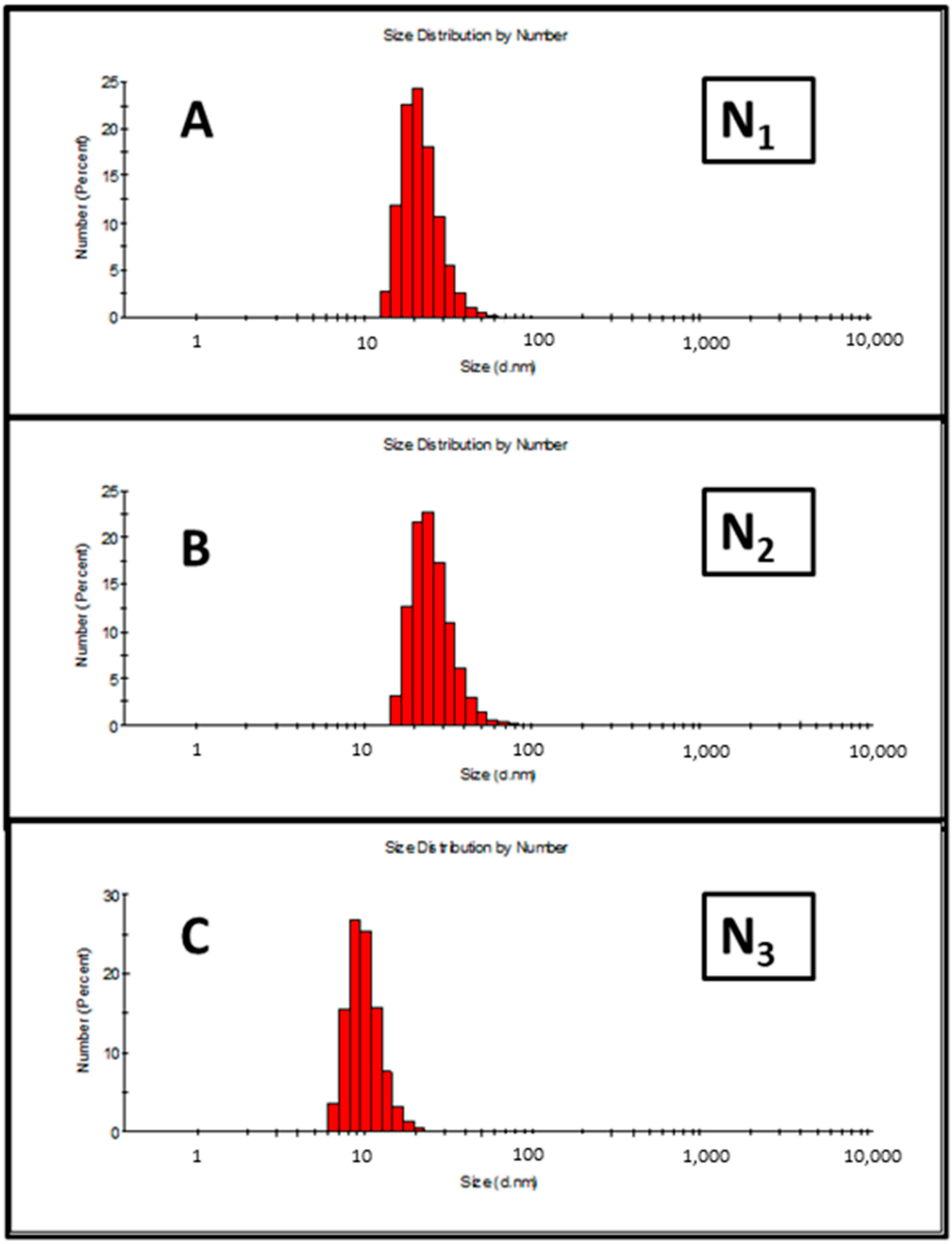

Figure S18. DLS size distribution by number of Au@16-Ph-16 nanoparticles at different $C_{Au@16\text{-}Ph\text{-}16}$ concentrations in PBS solution diluted at 0.1x concentration (ionic strength = 1.63 mM, PH = 7.4). The three Au@16-Ph-16/DNA-5-Fu formulations designated as C_i in abbreviated form. (**A**) C_1, $C_{5\text{-}Fu}$ = 0.25 µM, C_{DNA} = 0.30 µM, and $C_{Au@16\text{-}Ph\text{-}16}$ = 0.011 nM; (**B**) C_2, $C_{5\text{-}Fu}$ = 1.00 µM, C_{DNA} =1.19 µM, and $C_{Au@16\text{-}Ph\text{-}16}$ = 0.045 nM; and (**C**) C_3, $C_{5\text{-}Fu}$ = 7.5 µM, C_{DNA} = 8.9 µM, and $C_{Au@16\text{-}Ph\text{-}16}$ = 0.33 nM.

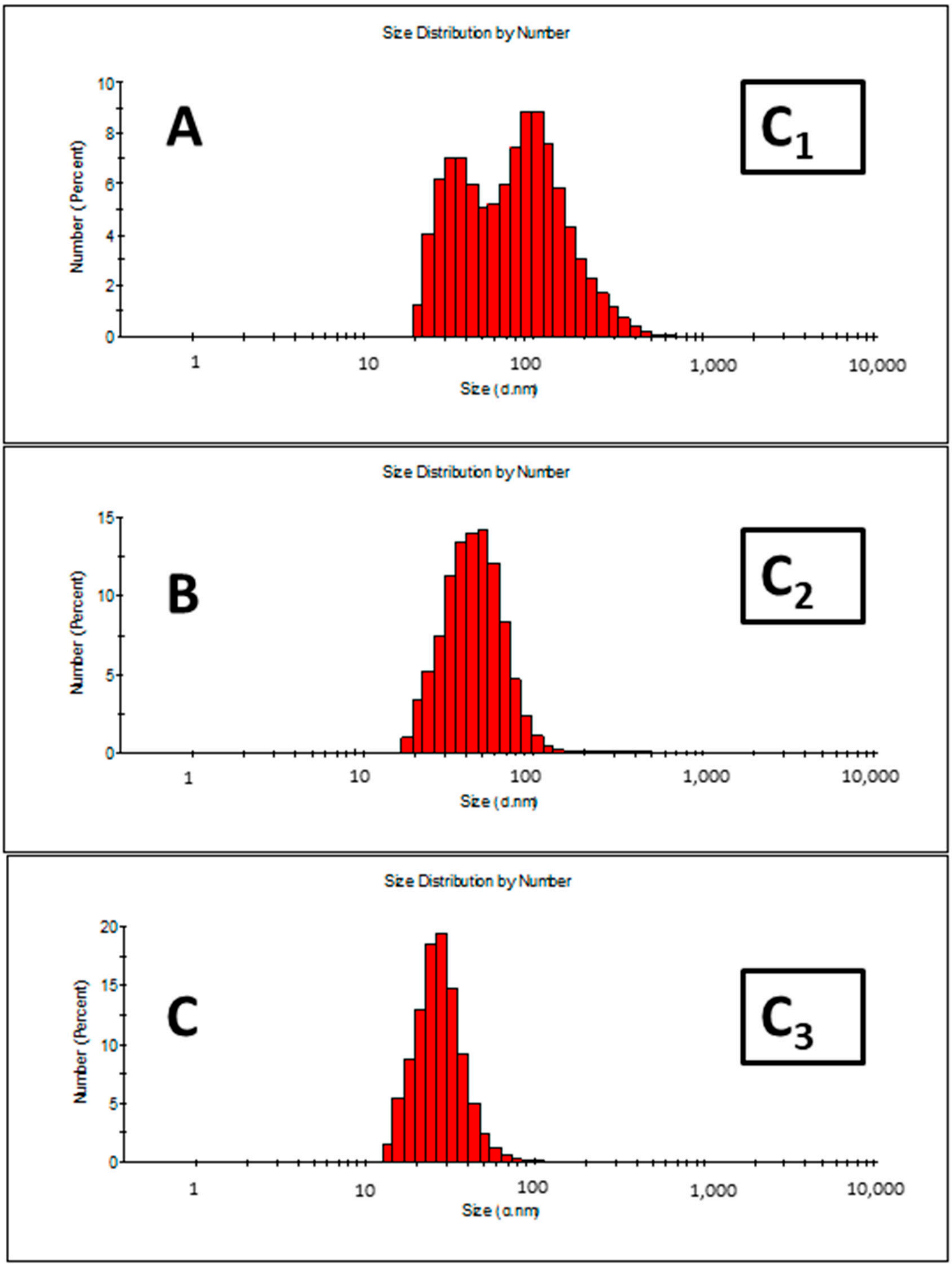

Figure S19. DLS size distribution by number of Au@16-Ph-16/DNA-5-Fu compacted nanosystems in PBS solution diluted at 0.1x concentration (ionic strength = 1.63 mM, pH = 7.4). The three Au@16-Ph-16/DNA-5-Fu formulations designated as C_i in abbreviated form. (**A**) C_1, $C_{5\text{-}Fu}$ = 0.25 µM, C_{DNA} = 0.30 µM, and $C_{Au@16\text{-}Ph\text{-}16}$ = 0.011 nM; (**B**) C_2, $C_{5\text{-}Fu}$ = 1.00 µM, C_{DNA} =1.19 µM, and $C_{Au@16\text{-}Ph\text{-}16}$ = 0.045 nM; and (**C**) C_3, $C_{5\text{-}Fu}$ = 7.5 µM, C_{DNA} = 8.9 µM, and $C_{Au@16\text{-}Ph\text{-}16}$ = 0.33 nM.

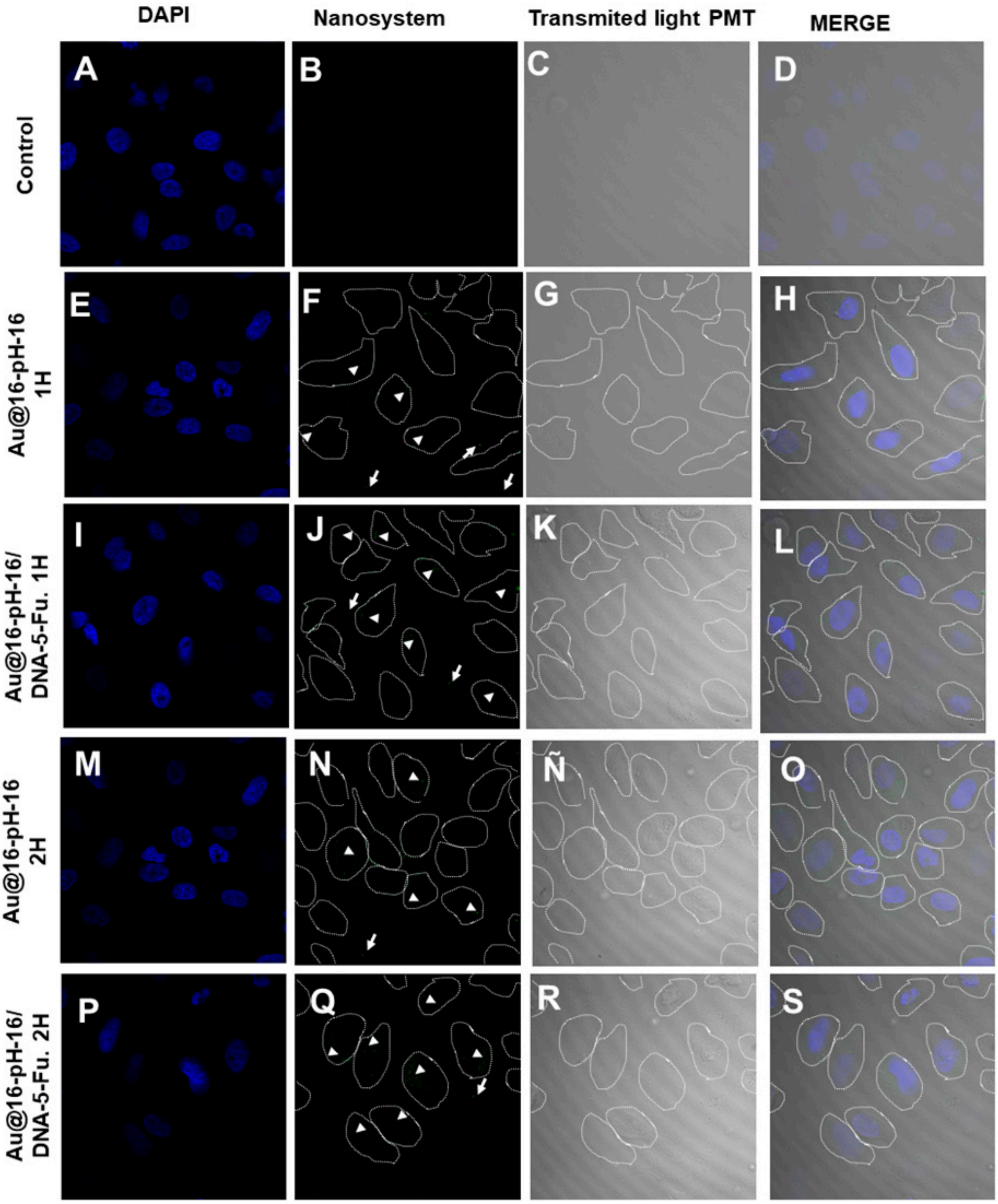

DAPI
Nanosystem
Transmited light PMT
MERGE
Control
Au@16-pH-16 1H
Au@16-pH-16/ DNA-5-Fu. 1H
Au@16-pH-16 2H
Au@16-pH-16/ DNA-5-Fu. 2H
A
B
C
D
E
F
G
H
I
J
K
L
M
N
Ñ
O
P
Q
R
S

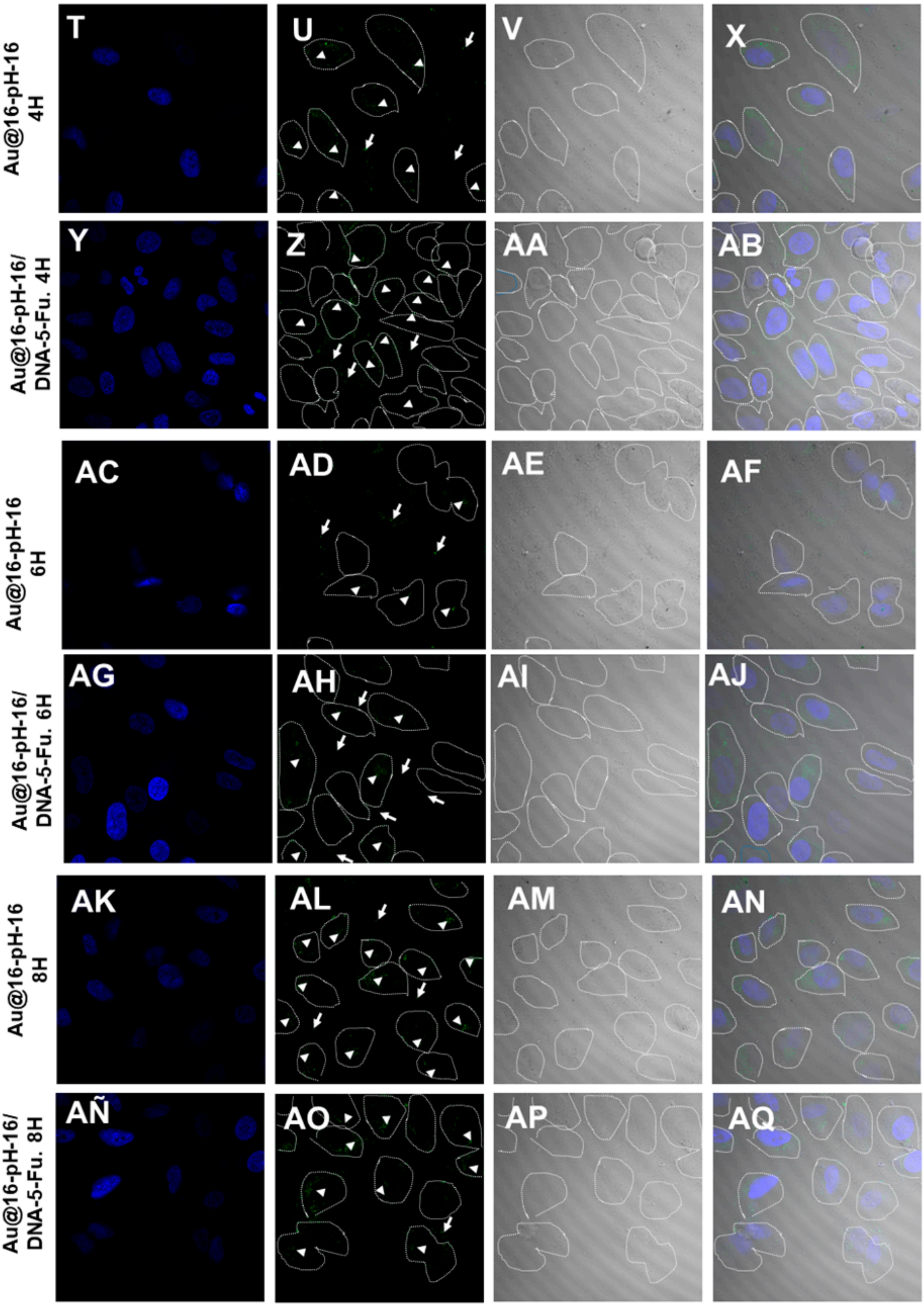
Au@16-pH-16 4H
T
U
V
X
Au@16-pH-16/ DNA-5-Fu. 4H
Y
Z
AA
AB
Au@16-pH-16 6H
AC
AD
AE
AF
Au@16-pH-16/ DNA-5-Fu. 6H
AG
AH
AI
AJ
Au@16-pH-16 8H
AK
AL
AM
AN
Au@16-pH-16/ DNA-5-Fu. 8H
AÑ
AO
AP
AQ

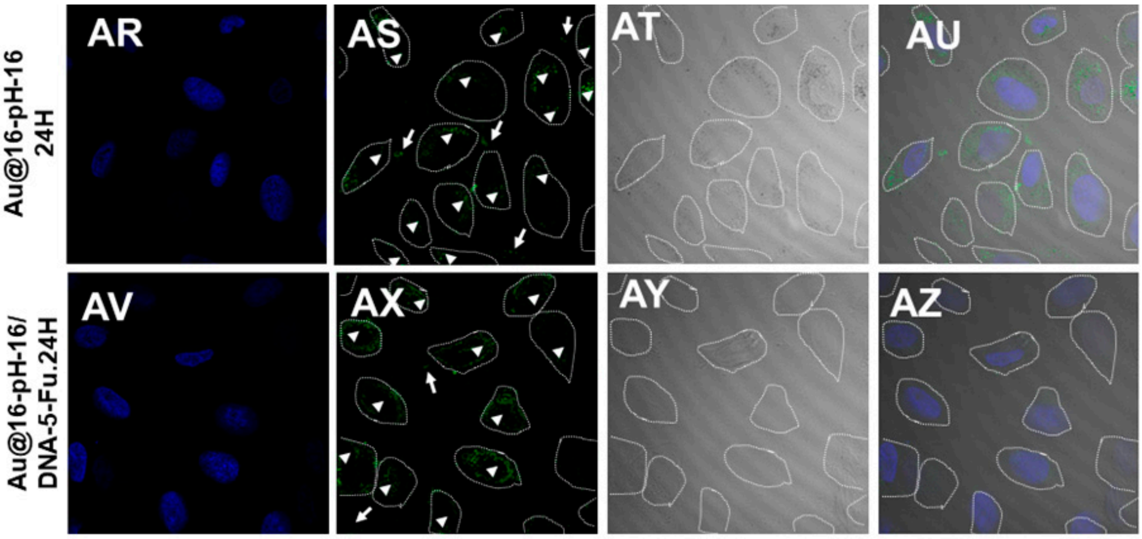

Figure S20. Confocal photomicrographs showing control of internalization of nanosystems in cells over the time. (**A**–**D**) Control, cells without treatment; (**E**–**H**) 1 h with Au@16-pH-16; (**I**–**L**) 1 h with Au@16-pH-16/DNA-5Fu; (**M**–**O**) 2 h with Au@16-pH-16; (**P**–**S**) 2 h with Au@16-pH-16/DNA-5Fu; (**T**–**X**) 4 h with Au@16-pH-16; (**Y**–**AB**) 4 h with Au@16-pH-16/DNA-5Fu; (**AC**–**AF**) 6 h with Au@16-pH-16; (**AG**–**AJ**) 6 h with Au@16-pH-16/DNA-5Fu; (**AK**–**AN**) 8 h with Au@16-pH-16; (**AÑ**–**AQ**) 8 h with Au@16-pH-16/DNA-5Fu; (**AR**–**AU**) 24 h with Au@16-pH-16; (**AV**–**AZ**) 24 h with Au@16-pH-16/DNA-5Fu. First column Cells labeled with DAPI; Second column presence or absence of N_3 or C_3 nanoparticles with fluorescence; Third column transmitted light; Fourth column shows the result of Merge. Dashed lines mark the cell contours. Arrows indicate nanoparticles on the cell exterior and arrow heads show nanoparticles internalized in cells.
